# Selection and characterization of a peptide-based complement modulator targeting C1 of the innate immune system†

**DOI:** 10.1039/d4cb00081a

**Published:** 2024-07-01

**Authors:** Sebastiaan M.W.R. Hamers, Leoni Abendstein, Aimee L. Boyle, Seino A.K. Jongkees, Thomas H. Sharp

**Affiliations:** a Department of Cell and Chemical Biology, Leiden University Medical Centre 2300 RC Leiden The Netherlands t.sharp@bristol.ac.uk; b Leiden Institute of Chemistry, Leiden University 2333 CC Leiden The Netherlands; c School of Chemistry, University of Bristol Bristol BS8 1QU UK; d Department of Chemistry and Pharmaceutical Sciences, Vrije Universiteit Amsterdam 1081 HV Amsterdam The Netherlands s.a.k.jongkees@vu.nl; e School of Biochemistry, University of Bristol Bristol BS8 1TD UK

## Abstract

The human complement pathway plays a pivotal role in immune defence, homeostasis, and autoimmunity regulation, and complement-based therapeutics have emerged as promising interventions, with both antagonistic and agonistic approaches being explored. The classical pathway of complement is initiated when the C1 complex binds to hexameric antibody platforms. Recent structural data revealed that C1 binds to small, homogeneous interfaces at the periphery of the antibody platforms. Here, we have developed a novel strategy for complement activation using macrocyclic peptides designed to mimic the interface between antibodies and the C1 complex. *In vitro* selection utilizing the RaPID system identified a cyclic peptide (cL3) that binds to the C1 complex *via* the globular head domains of C1q. Notably, when immobilized on surfaces, cL3 effectively recruits C1 from human serum, activates C1s proteases, and induces lysis of cell-mimetic lipid membranes. This represents the first instance of a peptide capable of activating complement by binding C1 when immobilized. Further characterization and synthesis of deletion mutants revealed a critical cycle size of cL3 essential for C1 binding and efficient complement activation. Importantly, cL3 also demonstrated the ability to inhibit complement-mediated lysis without affecting C1 binding, highlighting its potential as a therapeutic modality to prevent complement-dependent cytotoxicity whilst promoting cellular phagocytosis and cell clearance. In summary, this study introduces the concept of “*Peptactins*” – peptide-based activators of complement – and underscores the potential of macrocyclic peptides for complement modulation, offering potential advantages over traditional biologicals in terms of size, production, and administration.

## Abstract 2

Structure-inspired peptide design and RaPID selection identifies a macrocyclic peptide with immunotherapeutic potential, and inspires development of complement-modulating drugs.

## Graphical abstract

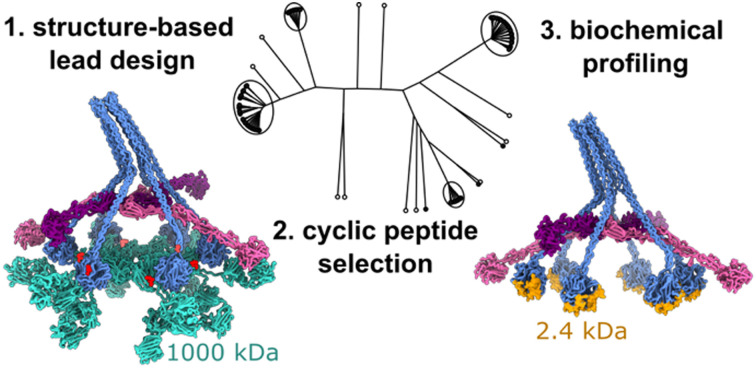

## Introduction

**Fig. 1:**
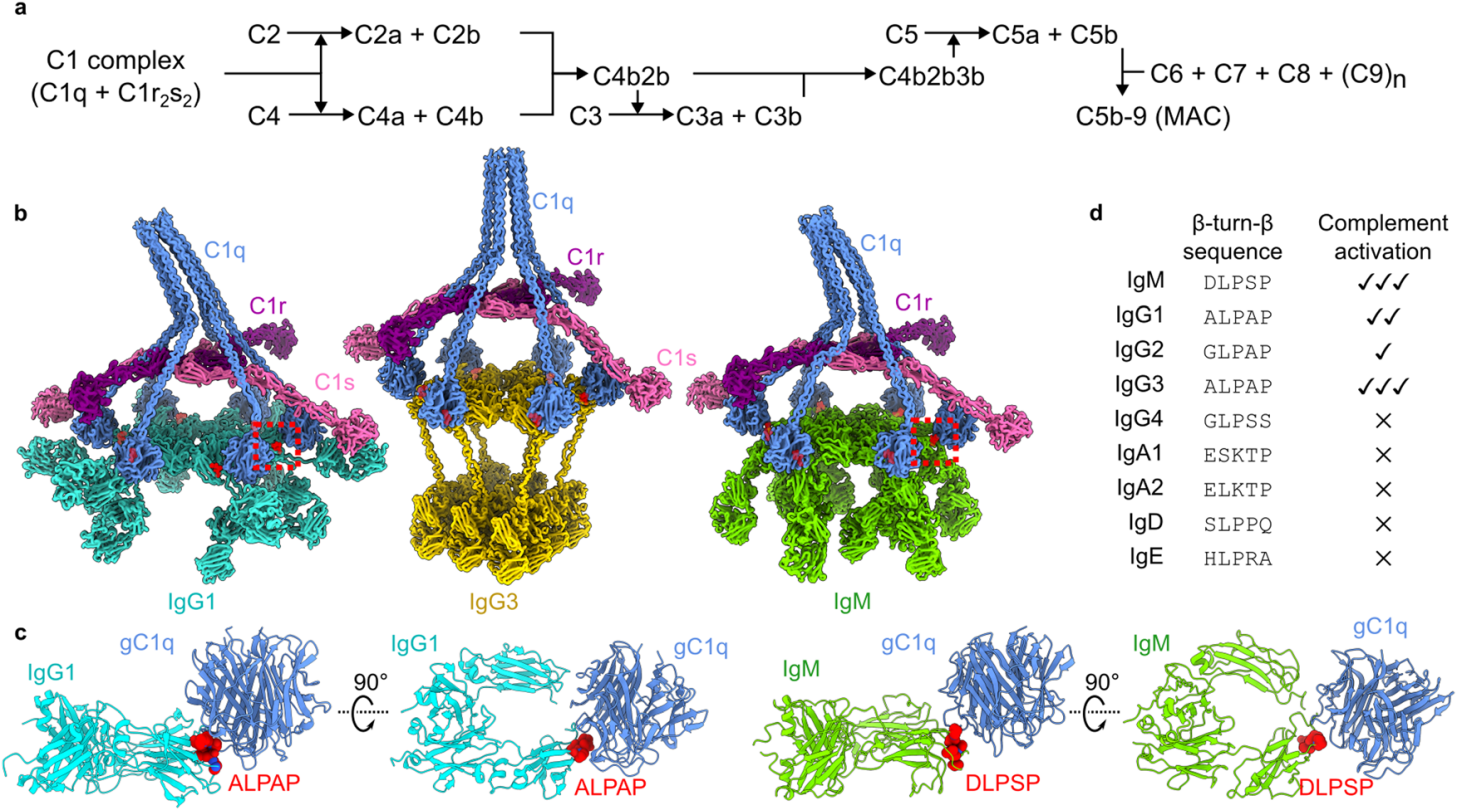
The classical complement pathway is initiated by defined structural motifs. (a) Overview of the classical complement pathway. (b) CryoEM-derived structures of antibody-C1 complexes. The interfaces between the gC1q domains and antibodies are highlighted in red. Structures were built into maps with accession codes EMD-4232, EMD-16241, and EMD-4878 (IgG1, IgG3 and IgM, respectively). (c) Structures of the interfaces (dashed boxes in (b)) between gC1q and ALPAP (IgG1 and IgG3; left) and DLPSP (IgM; right). (d) Sequence comparison of the β-turn-β motif of human antibody (sub)classes, and their ability to activate complement.^9^

The human complement pathway is part of our innate immune system and protects us against infections, but also mediates clearance of cellular debris and helps to regulate autoimmunity. The classical complement pathway can be initiated when the first component of complement, the C1 complex (Fig. 1a), binds to antibodies, specifically Immunoglobulin (Ig) M or IgG subclass 1, 2 or 3.^1^ IgM circulates as an obligate pentamer or hexamer,^2,3^ and IgG1 and IgG3, although monomers in solution, both form hexameric platforms upon antigen binding (Fig. 1b).^4,5^ The hexameric platforms comprise the antibody fragment crystallizable (Fc) regions, to which the C1 complex can bind.^6^ The C1 complex comprises the C1q ligand binding and scaffolding complex (composed of a hexamer of heterotrimeric proteins), which encapsulates the serine proteases C1r and C1s, that form a heterotetrameric C1r_2_s_2_ platform. Binding of the six globular head domains of C1q (gC1q) to the Fc platforms induces activation of C1r, which then cleaves C1s to form the active serine protease. Active C1 initiates a cascade of proteolytic events (Fig. 1a), as C1s proceeds to cleave the soluble proteins C4 and C2 to progress the complement pathway. The pathway deposits the opsonins C4b, C3b and C5b onto the surrounding surfaces, which bind to receptors on phagocytic cells to enhance phagocytosis.^7,8^ The pathway terminates with the formation of the membrane attack complex (MAC), which forms a pore that lyses the targeted membrane. Anaphylatoxins are also released upon cleavage of C4, C3 and C5, known as C4a, C3a and C5a, which attract phagocytic cells.

Complement is emerging as a focus for therapeutic intervention.^7,10^ In particular, complement-targeting macrocyclic peptides are being developed to block C1 activation,^11,12^ complement-mediated hemolysis^13^ and inflammatory signaling,^14^ which are being developed to treat hypoxic ischemic encephalopathy,^15^ paroxysmal nocturnal hemoglobinuria, and myasthenia gravis,^16^ respectively. These treatments are all antagonistic; they block complement activation or progression. In contrast, a new generation of therapeutics are being developed to activate complement as a route to achieve targeted cell killing *via* complement-dependent cytotoxicity (CDC).

CDC has been exemplified by antibody-based immunotherapeutics, such as Alemtuzumab, Daratumumab and Rituximab.^17–20^ This was seen as an adjunct of monoclonal antibody therapy. Recently, however, CDC has been actively pursued as a desired therapeutic mechanism on its own. Hexamerizing mutants of IgG comprise a platform of CDC-inducing immunotherapeutics,^21,22^ which are now being directed towards CD20 and CD52 for depletion of hematological cells. More recently, nanobodies that recruit C1 to specific cell types are being developed to target epidermal growth factor receptor (EGFR) and the HIV-1 envelope protein.^23,24^

Knowledge of the structural details and constraints of C1 binding and activation have been instrumental in these developments.^6,25^ The cryo-electron microscopy (cryoEM)-derived structures of antigen-bound to IgM, IgG1 and IgG3 all showed a common structural motif present during C1 binding (Fig. 1b).^2,4,5^ These data revealed that interaction between the 766 kDa C1 complex and the hexameric Fc platforms (900–1000 kDa) is mediated by small motifs at the periphery of the Fc domains (Fig. 1c).^2,4^ These structural motifs derive from homologous sequences in IgG and IgM (Fig. 1d), and contain a conserved LPxP (leucine, proline, glycine/serine, proline) sequence that adopts a β-turn-β hairpin at the periphery of the Fc platform. Although antibodies differ in their abilities to activate complement, as well as mediate other effector functions^9^, this motif is present in all human antibody (sub)classes that are able to activate complement (Fig. 1d).^1^

Here, we have taken inspiration from this structural motif to design and select macrocyclic peptides that mimic the interface between antibodies and gC1q, with the aim of developing a small molecule agonist that is capable of activating the classical complement system. Using the random nonstandard peptides integrated discovery (RaPID) system,^26–28^ we identified a cyclic peptide that binds to the C1 complex *via* the gC1q domains. In solution, this peptide can inhibit complement activation. Furthermore, when immobilised on surfaces, this peptide is capable of recruiting C1 from human serum, activating the C1s proteases, and inducing MAC pore formation on cell-mimetic lipid membranes. Competition assays have revealed a novel mechanism for complement inhibition that does not affect C1 binding. To the best of our knowledge, this is the first time a peptide has been developed that has been shown to activate complement by binding C1 when immobilised on surfaces.

## Results

### Selection of a macrocyclic peptide able to recruit C1q from human serum

**Fig. 2:**
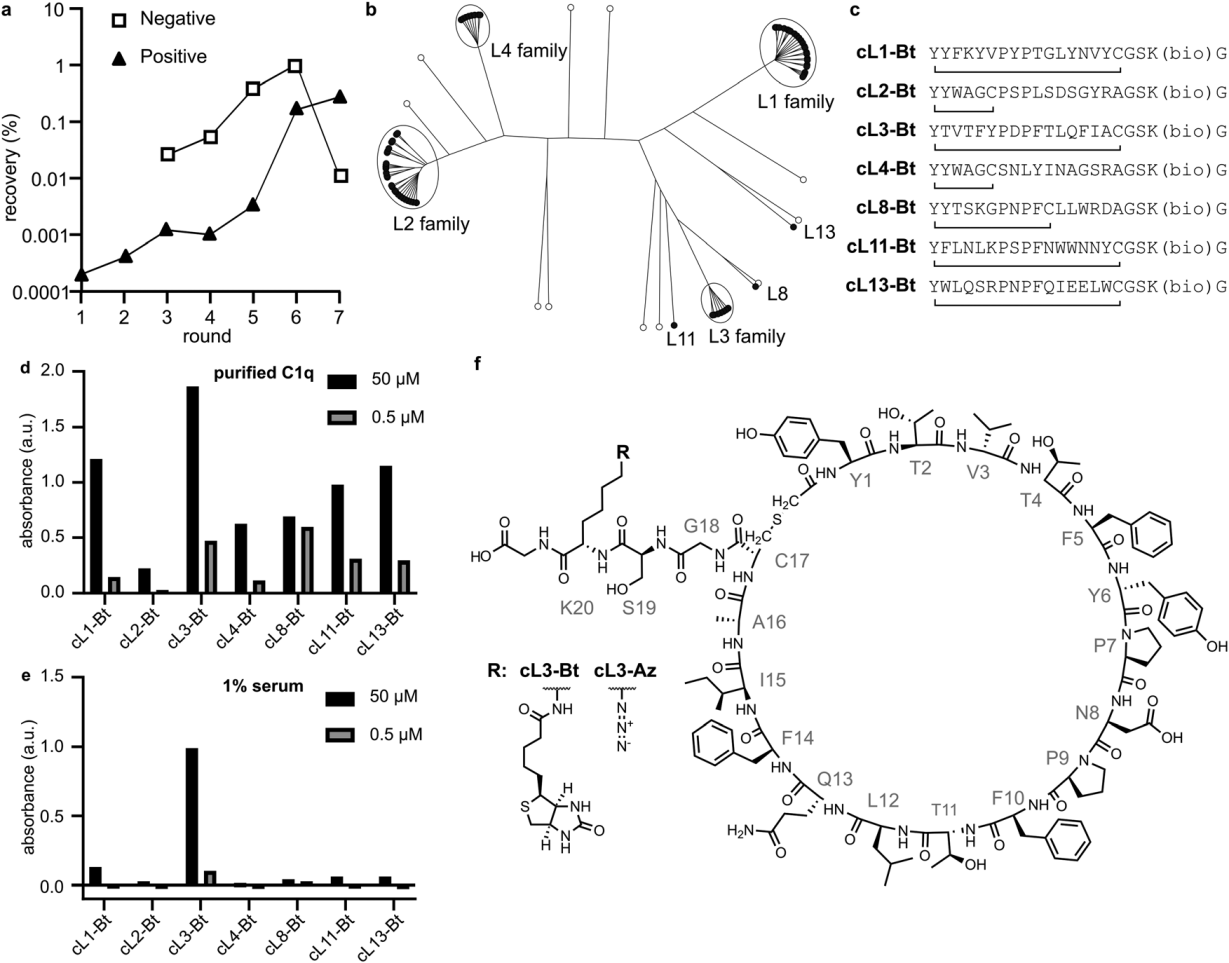
RaPID selection, sequence analysis and peptide screening. (a) qPCR quantifying DNA recovery after each selection round. cDNA conjugated peptides were either incubated with SCgC1q-presenting beads (positive) or beads without protein (negative). (b) Phylogenetic tree representation of the top 100 sequences. (c) Representative sequences obtained from each cluster in (b). Lines connecting N-terminal tyrosine and cysteine denote the position of cyclization, and (bio) denotes the C-terminal lysine analogue that was modified with biotin. (d) and (e) ELISAs where peptides were incubated with either purified C1q or human serum (1% in RPMI medium). Single datapoints are shown. Peptides were incubated at two concentrations 50 μM and 500 nM, either with purified C1q or 1% human serum. (f) Structure of cL3, which was modified with either a biotin (cL3-Bt) or azide (cL3-Az) moiety.

We immobilised a single-chain mutant of gC1q (SCgC1q) fused to a triple-step tag on streptactin-coated beads and used these to perform RaPID selection^26–28^ (Table S1, ESI†). NB; during RaPID selection, recombinant SCgC1q was used, however in all further experiments we use full-length C1q, and refer to gC1q as the globular domain of full-length C1q. In an attempt to mimic the β-turn-β structure of the native antibody motifs, we introduced obligate PxP codons into the mRNA library used for selection, resulting in 17-amino acid peptides with the sequence Y′x_5_PxPx_7_C, where x corresponds to any amino acid except methionine. Here, the N-terminal methionine has been recoded to an *N*-chloroacetylated-l-tyrosine residue (Y′), which cyclises with either the C-terminal cysteine residue, or a cysteine encoded within the random stretch of residues. The mRNA library therefore corresponds to ∼10^12^ potential different peptide sequences,^28,29^ which are tagged during *in vitro* translation with their encoding mRNA. These were panned against the immobilised SCgC1q with 7 rounds of positive selection, and 5 rounds of negative selection against beads without SCgC1q (Fig. 2a). The resulting library was subjected to next-generation sequencing and cluster analysis to identify peptides that bind with measurable affinity to gC1q. Analysis of the top 100 most abundant unique sequences identified 7 sequences or families that were selected by comparing relative abundance and intersequence differences (Fig. 2b).

For each family the most abundant sequence was chemically synthesized (Fig. 2c). For any sequences with two cysteines, the most C-terminal residue was mutated to an alanine, resulting in smaller macrocycles. These sequences were synthesised with a biotin on a C-terminal lysine sidechain after a GS linker, and the crude peptides were cyclised by reacting the N-terminal chloroacetylated tyrosine with the single cysteine residue. Biotinylated crude peptides were immobilised on a streptavidin-coated ELISA plate and recruitment of C1q analysed (Fig. 2d). Successful binding of full-length purified C1q was measured for all peptides, albeit with different efficiencies. However, analysis of C1q recruitment from human serum indicated that only cyclic-L3 (cL3) was able to efficiently bind (Fig. 2e and f).

### Surface-bound cyclic-L3 activates complement

**Fig. 3:**
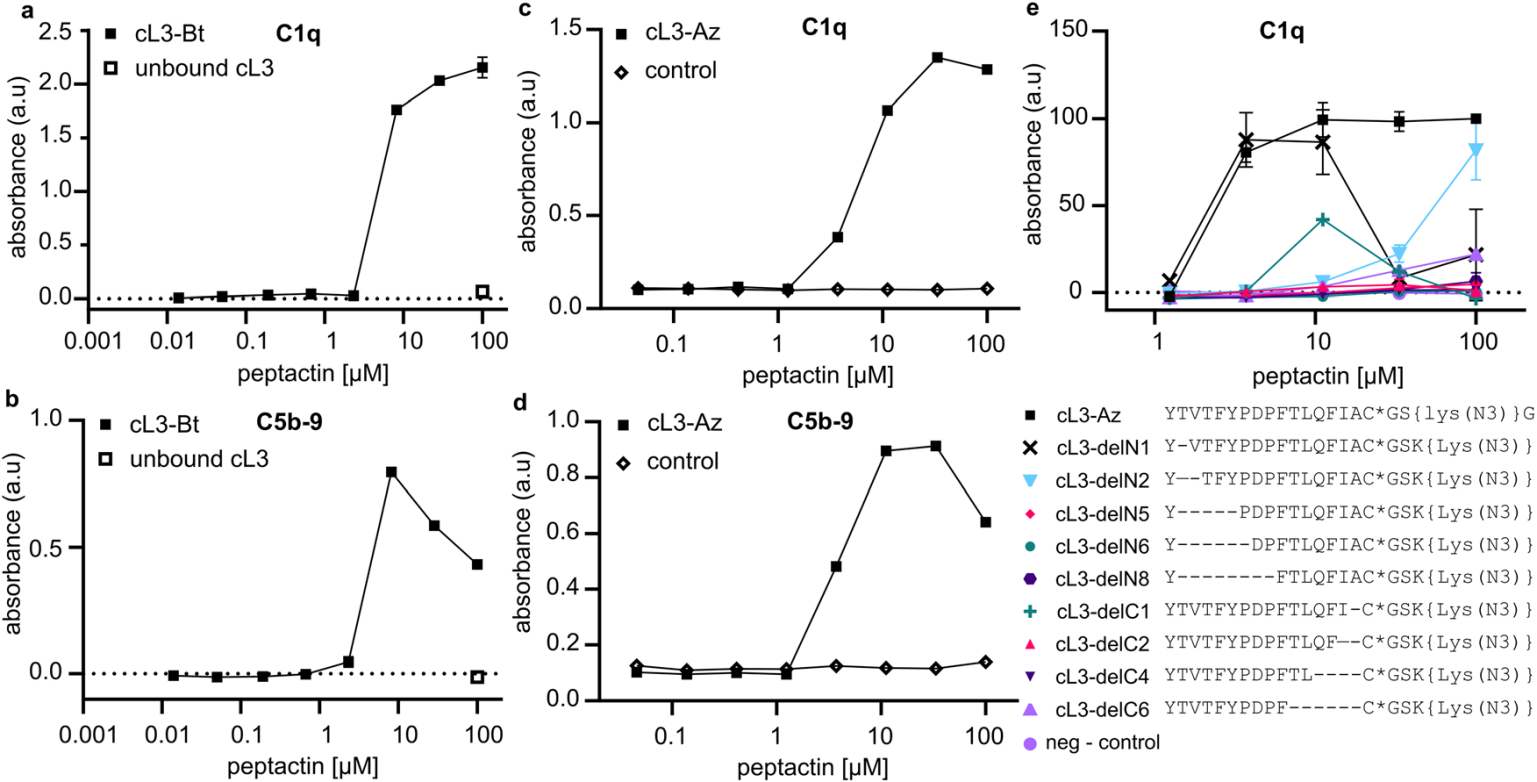
Activation of complement *via* C1q binding to cL3. (a) Representative ELISA detecting C1q was performed to evaluate the ability of cL3-Bt to bind C1 from 1% human serum in RPMI medium (two technical replicates). See Fig. S2 (ESI†) for fitted models of the data and additional technical replicates collected on different days. The control consists of unbound cL3, but no streptavidin, indicating the necessity of the streptavidin monolayer to recruit C1q. (b) ELISA detecting C5b-9 deposition to evaluate complement activation by cL3-Bt (two technical replicates). In both (a) and (b), streptavidin-coated plates are used and cL3-Bt is bound directly the biotin moiety. (c) As in (a), but this time the cL3-Az variant was immobilized on the plate using a DBCO-biotin linker that was co-incubated with the cL3-Az peptide on the plate for 1 hour at 37 °C. Thereafter, excess peptide was washed away and peptides were incubated with 1% human serum in RPMI medium before C1q was detected. Control is the condition with only biotin-DBCO linker present. See Fig. S4 (ESI†) for fitted model of the data. (d) As in (c), but this time with detection of C5b-9. (e) Deletion mutants of cL3 as in c: binding was assessed to determine recruitment of C1q from 1% human serum in PRMI. Lys(N3) denotes the lysine residue with the sidechain amine replaced by an azide, and * denotes that the cysteine has been cyclized *via* the N-terminal chloroacetyl modification. (c) and (d) display the data of one titration of cL3-Az. Further replicates of cL3-Az are contained in (e), which displays technical duplicates.

Peptactin cL3 was resynthesized at a larger scale and purified; see methods section for details. Purified biotinylated cL3 (cL3-Bt) (Fig S1 and Table S3, ESI†) was bound to a streptavidin-coated plate, but this time the ability of cL3-Bt to both recruit C1q from human serum and activate complement was assessed. Activation was measured by detecting C5b-9, which are the components of the terminal complement pathway that comprise the MAC (Fig. 1a), a pore that lyses lipid membranes. Again, cL3-Bt displayed efficient C1q recruitment, but this time also showed C5b-9 deposition (Fig. 3a and b), indicating successful C1 activation. This biotinylated cL3 molecule recruits C1 with EC50 values of ∼6 μM (Fig. S2, ESI†). This data demonstrates the ability of macrocyclic peptides to activate the human complement system, and so we have named this class of molecule “*Peptactin”*, for peptide-based activator of the complement system.

### Peptactin cL3 can activate complement on lipid membranes

Complement activation occurs on cell membranes so, to move towards more native systems, we produced liposomes to act as cell mimetics. Peptactin cL3 was now synthesised with an azide moiety (cL3-Az) in place of the biotin molecule on the lysine sidechain (Fig. 2f and Fig. S3, Table S3, ESI†). To test the functionality of this peptide, a biotin-dibenzocyclooctyne (DBCO) linker was immobilised on a streptavidin-coated ELISA plate and reacted with cL3-Az for one hour, after which the excess peptide was washed off and human serum added to the plate. Detection of bound C1q and deposited C5b-9 revealed successful complement activation (Fig. 3c and d). Furthermore, the EC50 of C1 recruitment of cL3-Az was comparable to the biotinylated variant (Fig. S4, ESI†).

**Fig. 4:**
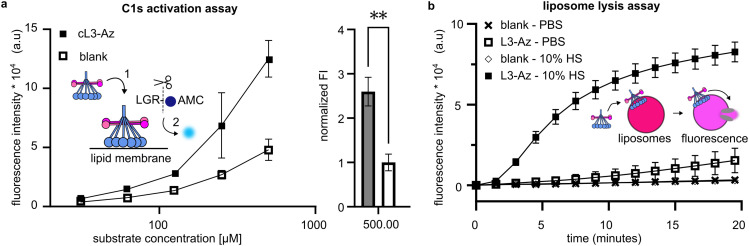
Activation of complement on lipid bilayer surfaces. (a) DBCO liposomes conjugated to cL3-Az can activate the C1s proteases. Activation of C1s^1^ results in cleavage of LGR-AMC and an increase in fluorescence.^2^ Raw fluorescence data is shown in the left. On the right, the fluorescence data with baseline activity of C1s in the presence of blank liposomes is set to 1, and displays a significant difference to the upregulated activity by cL3 modified liposomes (**, *p* < 0.01, unpaired *t*-test, Graphpad Prism version 9.3.1.) (One batch of conjugated liposomes, samples measured in triplicate). (b) DBCO liposomes conjugated to cL3-Az can be lysed *via* complement activation, resulting in an increase in fluorescence (conjugation was performed twice, on separate days and samples were measured in triplicate each time). Liposomes were exposed to 10% human serum (HS) in PBS and fluorescence was measured immediately after. Blank controls in (a) and (b) are liposomes that present 1% DBCO, but are not conjugated to cL3-Az.

Cell-mimetic liposome surfaces were then generated using a similar method; liposomes were synthesised including a lipid functionalized with a DBCO group displayed at 1 mol% on the surface of the membrane before cL3-Az was added. Liposomes now displaying cL3 were then purified before addition of pure C1 complex (composed of C1q, C1r and C1s). Binding of C1q leads to activation of C1r, which in turn activates C1s. Activated C1s is a serine protease that is responsible for propagating the complement pathway. To assess C1s activation, we added a non-fluorescent substrate, Boc-Leu-Gly-Arg-Amino Methyl Cumarin (LGR-AMC), which becomes fluorescent upon cleavage by C1s.^30^ Membrane-bound cL3 was able to significantly activate C1s when compared to liposomes without cL3 (Fig. 4a).

Next, the ability of cL3 to activate complement and cause membrane lysis *via* formation of the MAC pore was assessed. Liposomes were synthesised displaying DBCO as above, but this time were formed encapsulating a high concentration of sulforhodamine B, which is self-quenched and non-fluorescent. Upon complement activation and MAC pore formation, the sulforhodamine B becomes diluted and fluoresces. Purified liposomes containing sulforhodamine B and displaying cL3 were mixed with human serum and the fluorescence monitored. A fluorescence increase was seen only on liposomes displaying cL3 and in the presence of human serum (Fig. 4b), indicating that peptactin cL3 is able to activate complement and cause MAC pore formation on lipid bilayers.

### Deletion mutants of peptactin L3 determine the structural constraints of the macrocycle

The macrocycle of cL3 is relatively large as compared to other macrocyclic drugs.^31^ Therefore, systematic deletions from cL3 were implemented to alter the ring size and determine which residue positions were important for gC1q binding and complement activation. Seven deletion mutants were synthesised by removing up to 8 residues from the N- and C-termini (not including the N-terminal tyrosine or C-terminal cysteine; Fig. 3e and Fig. S5–S13, Table S3, ESI†), and their ability to bind to C1q in human serum was determined. Removal of one amino acid from the N-terminus (cL3-delN1), which corresponds to threonine2, did not appear to affect C1q binding at low concentration, but caused reduced binding at higher concentrations, indicating a slight disruption compared to cL3. However, removal of two residues (cL3-delN2) caused much weaker binding. Removal of one residue from the C terminus (cL3-delC1) also caused much weaker binding, whilst any other deletion seemingly abolished C1q binding.

### Solution-phase cL3 can inhibit complement activation

**Fig. 5:**
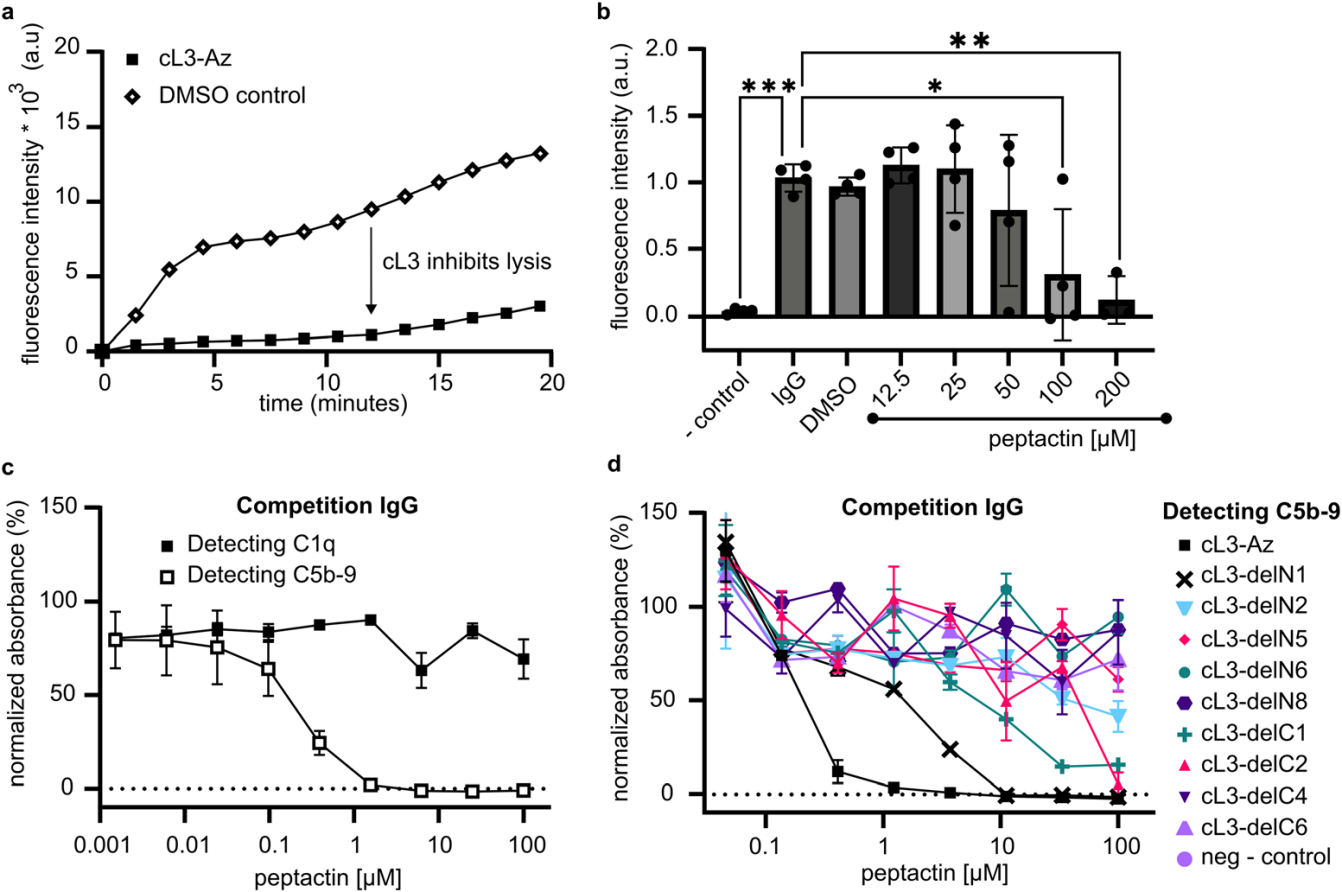
Inhibition of antibody-dependent complement activation by cL3. (a) The ability of cL3 to inhibit complement activation by IgG antibody platforms was assessed. Human serum, 10%, was incubated with 100 μM of cL3 10 min, and fluorescence increase caused by liposome lysis was monitored. (one representative experiment of data shown in (b)). (b) A titration of cL3 was performed under the same conditions as (a) (*n* = 4 technical replicates). A one-way ANOVA, followed by a Tuckey test was performed comparing the fluorescent data after 20 minutes to the IgG condition (*p* < 0.05 *, *p* < 0.01 **, *p* < 0.001 ***). (c) Competition ELISAs, performed in 1% human serum, determining cL3 mediated inhibition of C1q and C5b-9 deposition by pooled IgG, using cL3-Az. Samples are normalized to the DMSO control (maximal signal, 100%) and no serum samples (minimal signal, 0%) (two technical replicates). (d) Competition ELISAs as in (c) determining inhibition of C5b-9 deposition by cL3-deletion mutants (two technical replicates).

The classical complement pathway is initiated upon C1 binding to ligands, which include IgG antibodies, and it is known that C1 modulators can display both inhibitory and agonistic properties depending on how these molecules act in solution.^23,25^ The ability of peptactin cL3 to inhibit complement activation was investigated further using antigenic liposomes. Liposomes were prepared displaying antigens based on a CD52 mimotope.^2^ CD52-presenting liposomes were synthesized containing self-quenching sulforhodamine B. Upon addition of anti-CD52 IgG1 and human serum to these liposomes, complement activation occurs, as measured by an increase in fluorescence upon MAC pore formation (Fig. 5a). However, preincubating the serum with 100 μM of cL3 for 10 minutes results in statistically-relevant inhibition of complement-mediated liposome lysis (Fig. 5a). Subsequently, a titration of cL3 was performed, giving an approximate IC50 of ∼65 μM (Fig. 5b and Fig. S14, ESI†).

To determine how peptactin cL3 was inhibiting complement activation, pooled human IgG was coated on ELISA plates and the ability of cL3 to inhibit either C1q binding or C5b-9 deposition was assessed (Fig. 5c). Although cL3 was not able to inhibit C1q binding to pooled IgG, it did inhibit complement activation, as measured by reduced C5b-9 deposition, with an IC50 of ∼0.25 μM in this system (Fig. S15, ESI†). The cL3 deletion mutants were also evaluated for their capacity to inhibit 5b-9 deposition (Fig. 5d). As before for the C1q recruitment data (Fig. 3e), any deletion was detrimental for inhibition, but here we observed maximal inhibition with full-length cL3, followed by cL3-delN1, cL3-delC1, and cL3-delC2, respectively.

**Fig. 6:**
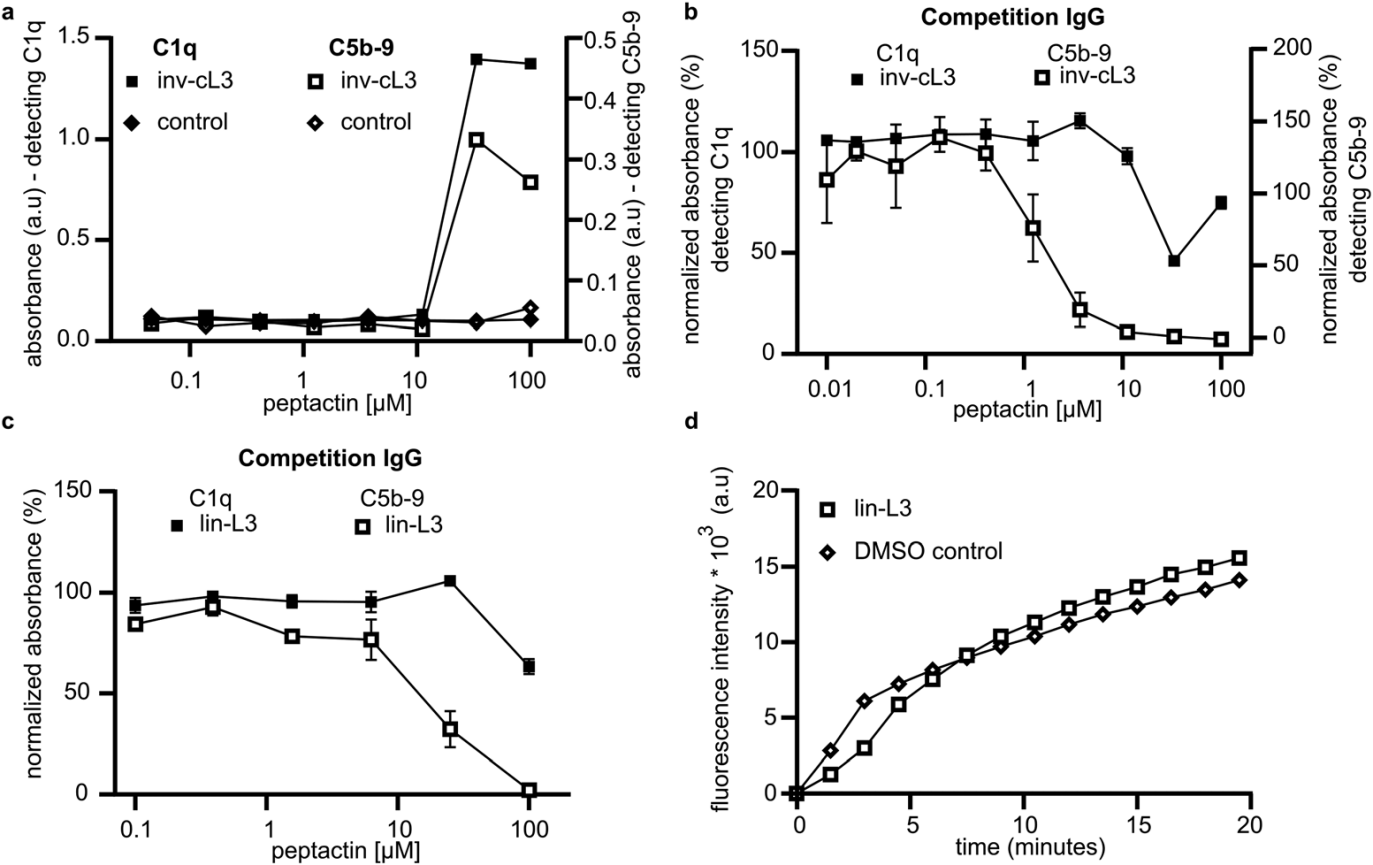
cL3 variants effect on complement modulation. (a) ELISA determining the activation of complement in 1% human serum by azide modified inv-cL3 detecting C1q and C5b-9 (technical singlet). (b) Competition ELISA showing inv-cL3 inhibiting C1q and C5b-9 deposition by blocking binding to pooled IgG (two technical replicates). (c) Competition ELISA showing lin-cL3 inhibiting C1q and C5b-9 deposition by blocking binding to pooled IgG (two technical replicates). (d) The ability of lin-L3 to inhibit complement activation by IgG was assessed in liposome lysis assays (single experiment shown, experiment was repeated twice, once at 10 min pre-incubation, once at 1 hour).

We utilised this competition assay to determine the importance of other structural features of cL3 by synthesising analogues of cL3. Peptactin cL3 is cyclised between the N-terminal chloroacetyl group and the cysteine residue at position 17 (Fig. 2f). To determine the importance of the conformational state of L3 on C1q binding and complement activation, peptactin L3 was synthesised with the N-terminal l-form tyrosine residue exchanged for a d-isomer, henceforth named inv-cL3 (Fig. S16 and Table S3, ESI†). Changing this stereochemistry has been previously shown to impact the efficacy of macrocyclic peptides.^32,33^ Inv-cL3 was assessed for its agonistic properties, revealing an approximate 2-fold decrease in EC50 compared to cL3 (Fig. 6a and Fig. S17, ESI†). Peptactin L3 is therefore more potent when composed entirely of l-form amino acids. Next, the ability of inv-cL3 to inhibit C1q binding to pooled IgG was determined. Here, inv-cL3 demonstrated minor inhibition of C1q binding to pooled IgG at high concentrations (Fig. 6b), but more apparent was the ability of inv-cL3 to inhibit C5b-9 deposition, which was inhibited with an IC50 of ∼1.5 μM (Fig. 6b and Fig. S18, ESI†). This was significantly worse than full-length cL3, and in line with activation experiments. Finally, we synthesised a linear variant of L3 (lin-L3) by replacing cysteine17 with a serine, and omitting the chloroacetyl group during synthesis (Fig. S19 and Table S3, ESI†). Lin-L3 displayed a slight reduction in C1 binding to pooled IgG, and C5b-9 deposition (Fig. 6c), but was much less effective than cL3 or inv-cL3. Furthermore, lin-L3 was not able to inhibit MAC pore formation, even after preincubating the serum with 100 μM of lin-L3 for 1 hour (Fig. 6d).

### Mapping the binding site of cL3 onto gC1q

**Fig. 7:**
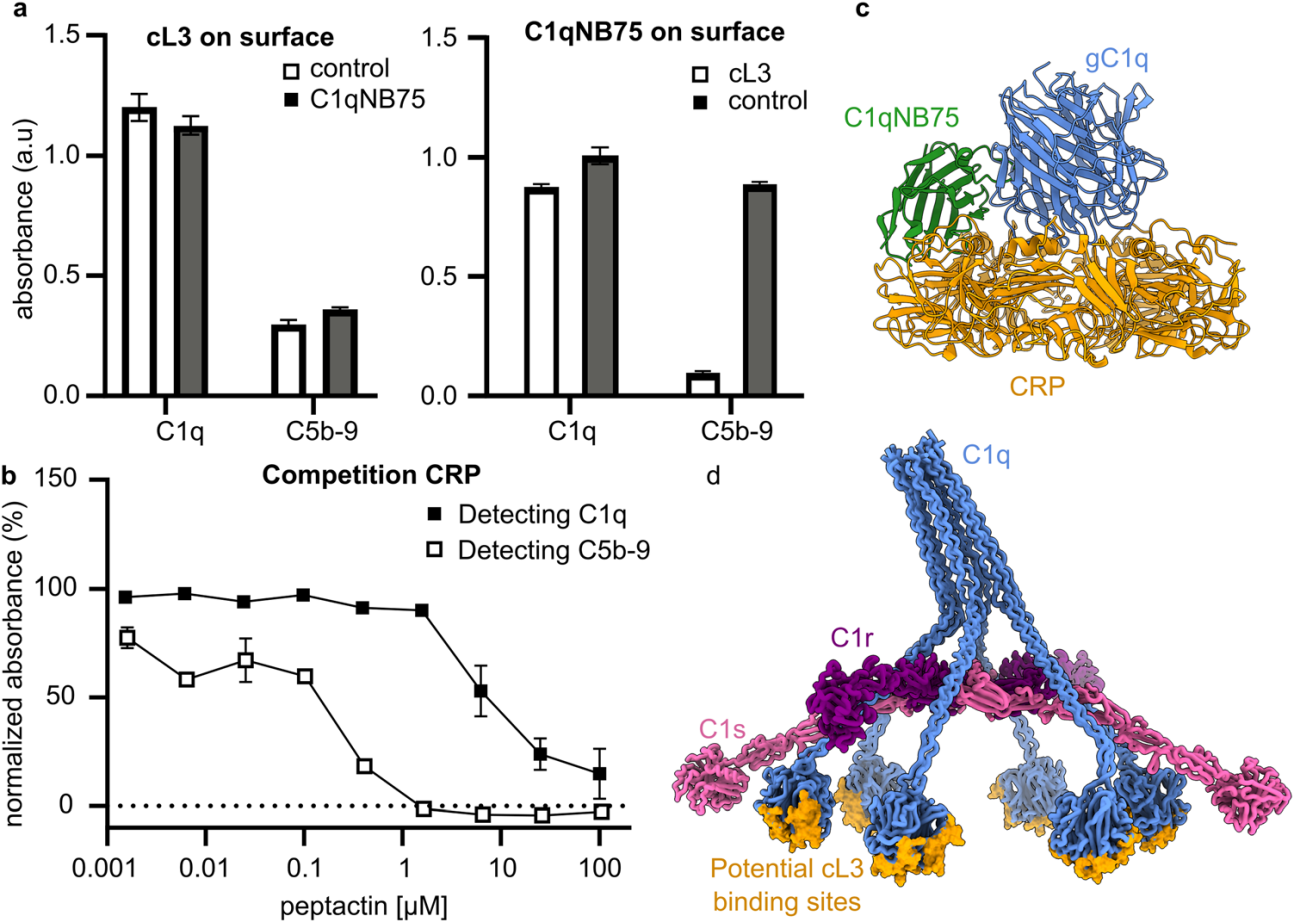
Competition assays between cL3 and C1q binders. (a) Competition ELISAs where either cL3-Bt (left) or C1qNB75 (right) are immobilised and competed against solution phase C1qNB75 (100 nM) or cL3 (20 μM), respectively, in the presence of 1% human serum. Binding of complement component C1q and deposition of C5b-9 was measured. (Three technical replicates) (b) ELISAs determining cL3-mediated inhibition of C1q binding and C5b-9 deposition by CRP. 1% human serum was incubated with cL3-Az before adding to the wells containing CRP. Samples are normalized to the DMSO control (maximal signal, 100%) and no serum samples (minimal signal, 0%). (Two technical replicates) (c) binding locations of CRP (orange) and C1qNB75 (green) on gC1q (blue). (d) C1 complex showing potential cL3 binding locations (orange).

To gain insights into the binding location on gC1q, competition assays were performed with C1 ligands and binders. The nanobody C1qNB75 binds gC1q at a known position^25^, and can therefore be used to help map the binding site of cL3 on C1q. A competition ELISA, using immobilised cL3 and solution-phase C1qNB75 (Fig. S20, ESI†), showed that cL3 does not compete with C1qNB75 (Fig. 7a). However, when C1qNB75 was immobilised instead of cL3, solution-phase cL3 showed inhibition of only C5b-9 deposition but not C1 binding (Fig. 7a), similarly to IgG (Fig. 5c).

As well as being initiated by antibodies, the classical complement pathway can also be activated upon C1 binding to the pentraxin C-reactive protein (CRP).^34,35^ The binding site of gC1q to CRP has been determined using a combination of mutagenesis studies and cryo-electron tomography,^36,37^ and so this can be used to determine possible binding locations of cL3 to gC1q. CRP was coated on ELISA plates and the ability of cL3 to inhibit either C1q binding or C5b-9 deposition was assessed. This revealed that cL3 was able to inhibit CRP-mediated complement activation *via* both reduced C1q (though at higher concentrations) binding and C5b-9 deposition, with the latter displaying an IC50 of ∼0.25 μM (Fig. 7b and Fig. S15, ESI†). This IC50 was comparable to the IgG inhibition, indicative of a mode of action that is irrespective of the ligand.

Together, these data imply that cL3 binds in the region proximal to CRP, at the bottom of the gC1q domain,^37^ and distal to the region where both IgG-Fc domains and the C1qNB75 bind (Fig. 7c), resulting in a map of potential cL3 binding sites on the complete C1 complex (Fig. 7d).

## Discussion

Therapeutic development of C1 agonists has mainly focussed on biologicals. Engineered antibodies and nanobodies have shown promise for several clinical targets and some of these protein engineering techniques, such as the development of the Hexabody platform, are now under clinical evaluation.^21,23^ However, biologicals have inherent downsides, mainly due to their size, which pose challenges in administering to patients and costly production. Synthetic drugs such as peptides and small molecules offer solutions to these disadvantages. CryoEM structures of various antibodies in complex with C1 revealed that, while antibodies assemble into large ∼900–1000 kDa platforms, the actual interface of the platforms with gC1q comprised small homologous peptide sequences, consisting of ∼5 amino acids (Fig. 1).^2,4,5^ We therefore pursued a strategy to mimic these interfaces by utilising peptide design and selection. Here, we present a proof of principle that peptides can be used to recruit and activate C1 of the complement system. We introduce the term “*Peptactin*”, or peptide activator of the immune system, for peptides that fall into this category. By combining RaPID selection with synthetic biology methods, we provide a starting point for development of complement-activating drugs that target C1.

RaPID selection was used to obtain peptides that enable complement activation. For this reason, we introduced two conserved prolines found in the native C1-antibody interface, within a PxP motif, into the peptide library (Fig. 1d). The resulting peptides all displayed the ability to bind pure C1q (Fig. 2d), but only one, cL3, was capable of doing so in human serum (Fig. 2e). Presumably, the other peptides bind non-specifically to other proteins or components found in human serum, which then blocks recruitment of C1q. Synthesis of cL3 modified with either a biotin or an azide at the C-terminus indicated that the peptide was amendable for modification and targeting (Fig. 3). In particular, the azide moiety can be used for conjugation of diverse molecules and proteins *via* click-chemistry, such as potential drug delivery strategies for administering the peptactin^38^. Displaying cL3 on cell membrane mimetics led to efficient complement activation in the presence of human serum (Fig. 3 and 4), resulting in the first peptide-based complement agonist.

Deletion mutants were synthesised to explore the impact of the composition and size of the macrocycle. Deletion of one amino acid from either end of the peptide led to reduced binding and activation (Fig. 3e and 5d), indicating that these residue positions are important for binding, either *via* direct interaction with gC1q, or due to a necessity for the macrocycle to be 17 residues in length. Deletion of more than two residues abolished binding, but single deletions, from either the N- or the C-termini, were still able to activate complement, albeit with much reduced activity compared to the full-length cL3 (Fig. 5d). To determine whether tyrosine1 or cysteine17, which are both crucial for cyclisation of the peptide, are also involved with binding, we synthesised variants without cysteine (which then formed a linear peptide; lin-L3), or with l-tyrosine1 exchanged for d-tyrosine1 (inv-cL3). Both of these were worse than cL3 (Fig. 5), indicating that the peptactin is more effective when cyclised, but also that the tyrosine may be important for binding. However, changing amino acid stereochemistry has been shown to impact peptide structure^39^, which may account for the worse binding observed here.

We next evaluated the ability of cL3 to inhibit complement activation by performing competition assays against C1q binders. Peptactin cL3 can both activate and inhibit complement. Note that for complement activation two binding actions have to occur; binding to the target surface and binding to C1. To achieve inhibition, binding to solution-phase C1 can be sufficient to inhibit complement. Such divergent behaviour is also observed with C1qNB75, a nanobody that can both inhibit and activate complement *via* C1 binding depending on the context in which it is used.^23,25^ By incubating human serum with cL3, we could successfully reduce complement-mediated liposome lysis by inhibition of C5b-9 deposition (Fig. 5a and b). Surprisingly, however, C1q binding was not inhibited (Fig. 5c). These data indicate that C1q binding to IgG is not affected, but complement activation is inhibited. IgG is the native ligand for C1, and activates complement on cells by forming hexameric Fc platforms.^6^ A possible explanation for this is that cL3 is unable to inhibit all 6 headgroups of hexameric C1q from binding to IgG, therefore we see no inhibition of C1 binding as some of the gC1q domains are still available to bind. However, if cL3 does bind to some of the 6 gC1q headgroups, it may inhibit complement activation without inhibiting C1q binding, as observed here. Alternatively, it has been posited that a structural rearrangement may occur after C1q binding to IgG, but before C1r and C1s activation.^2,4,40^ It is conceivable that cL3 binds to a region on gC1q and impedes rotation of gC1q relative to the C1q collagen arms, which has been implied from antibody binding studies that identified a cryptic epitope accessible only on antibody-bound C1q.^40^ Peptides derived from peptide inhibitor of complement C1 (PIC1) also do not inhibit C1 binding to its native ligand, but instead prevent C1s activation.^12,41^ Interestingly, inhibition of C5b-9, but not C1 binding, is also observed for the nanobody C1qNB75 (Fig. 7b and c). C1qNB75 is known to bind at the same location as antibodies,^25^ and so the ability of cL3 to inhibit only complement activation, and not C1 binding, is conserved for other C1q binders. In contrast, cL3 inhibited C1q binding to CRP as well as complement activation, suggesting overlap in the binding region of CRP and cL3 (Fig. 7a). This is likely due to the binding site(s) for C1q to CRP being different to that of IgG (Fig. 7d).^37^

Complement inhibition has been extensively explored as a therapeutic route over the past decade; indeed, six complement inhibitors are now approved for clinical use.^13,42–46^ Two of these inhibitors target the C1r and C1s proteases,^42,43^ including administration of native human C1-inhibitor (C1-INH) to treat hereditary angioedema,^42^ or the humanized monoclonal antibody Sutimlimab to treat cold agglutinin disease.^43^ Similarly, PIC1 also inhibits the binding of the C1 proteases to C1q,^12^ and this family of inhibitors are currently being investigated in animal models and clinical trials as a treatment for acute and delayed hemolytic transfusion reactions, cystic fibrosis and diabetic wound healing.^41,47^ To the best of our knowledge, peptide 2J is the only peptide inhibitor of complement that has been developed to bind to the gC1q domains.^48^ In contrast to peptactin cL3, peptide 2J inhibited binding of C1q to IgG, leading to reduced complement activation. From the available data, peptactin cL3 compares favourably to these other peptide-based complement inhibitors in terms of IC50. Peptactin cL3 could therefore be used to expand the complement therapy toolbox, and initial tests performed here showcase potential to prevent CDC *via* a novel route of complement inhibition without affecting C1 binding (Fig. 5a and c). C1 is a ligand for complement receptor 1, which mediates opsonophagocytosis; clearance of tagged material by professional phagocytic cells.^49^ The ability of cL3 to allow binding of C1 to IgG-coated cells without activating complement may also therefore be a method to promote clearance of material *via* phagocytosis without inducing the inflammatory complement cascade.

Improving complement activation has been achieved by glycoengineering,^50,51^ antibody isotype engineering,^52,53^ Fc engineering,^54,55^ and enhancing IgG hexamerization.^6,21,22^ Indeed, it is likely that all of these approaches affect antibody oligomerization and the formation of an Fc platform to which C1 can bind. We envision cL3 being conjugated to targeting molecules such as nanobodies,^56^ Fab domains,^57^ peptides or small molecules, thereby creating a bispecific construct that can target clinically-relevant epitopes to which complement can then be recruited. As well as development of therapeutics, peptides such as cL3 may offer a method to expand our fundamental understanding of C1 activation. Cause-and-effect relationships of C1 binding to antibody platforms can be hard to isolate, since both antibody assembly and C1 binding must occur prior to activation. This adds a level of abstraction, which makes data interpretation complex. Furthermore, protein engineering can be labour intensive and costly; peptide synthesis overcomes those disadvantages. A monomeric binder such as cL3 could yield additional insights into the additive effect of ligand affinity and avidity, providing valuable data into the intricacies of C1 activation, thereby augmenting the design and development of complement-modulating therapies.

## Materials and methods

### RaPID selection on gC1q

Selections were performed on streptactin XT magnetic beads (Iba Lifesciences), which were used to immobilize purified single chain C1q (SCgC1q) modified with a triple Strep-tag, ordered from uProtein Express BV (Netherlands) (see Table S1 for the SC-GC1q amino acid sequence, ESI†).^26,58^ SCgC1q was produced in HEK293E-253 cells as previously described.^58^ Procedures of the selection are described in detail in Thijssen *et al*.^28^ Briefly, a DNA library was ordered from IDT (USA), which encoded a semi-random library (X_2_…X_6_P_7_X_8_P_9_X_10_…X_1_) of NNK codons. *N*-Chloroacetyl-l-tyrosine cyanomethyl esters were charged with enhanced flexizyme (‘eFx’) on tRNAcau (25 μM) such as described in Goto *et al.*^59^. For the selection, MagStrep® type3 Strep-Tactin®XT beads (Iba Lifesciences) were used. SCgC1q was co-incubated with the beads at a ratio of 400 ng per 1 μL of beads. Beads were washed using phosphate buffered saline (PBS) plus 0.1% Tween-20 prior to incubation and incubation was performed at 4 °C for 30 min under continuous rotation to keep resuspending the beads. The peptide sequence contains the modified tyrosine at position 1, and a cysteine residue at position 17, which is followed by a GSGSGS linker. RaPID selection comprises a series of sequential rounds of (i) RNA generation, (ii) peptide synthesis, in which the peptides are covalently linked to the RNA encoding them *via* a puromycin linker, (iii) generation of cDNA to the RNA tag on the peptide to stabilize the complex (iv) exposing library to SCgC1q in in phosphate buffered saline (PBS) plus 0.1% Tween-20 for 30 min at 4 °C, (v) washing and (vi) QC of the selection *via* qPCR. The pool cDNA that remained after binding to SCgC1q was amplified and subjected to another round of selection. After two rounds with only positive selection, a negative selection was performed where peptides were exposed to beads without bound SCgC1q for the remainder of the selection procedure until 7 rounds were performed in total. The negative selection was performed using blank beads and the library was exposed to the beads for 10 min at 4 °C under continuous agitation and then washed off. This procedure was repeated three times before exposing to the positive selection beads bound to SCgC1q. Additionally during the positive selection monomeric streptag peptide was added to the solution (30 mM) to block any free streptag binding sites and prevent selection of streptag-like peptides. Recovery percentages, as shown in the results section, are the percentage of output sample compared to sample input, as determined by qPCR. Beads containing the bound peptides were incubated in 50 μL of MQ (ultrapure water) at 95 °C for 5 min. The supernatant was removed and diluted 20 times in MQ of which 1 μL was used for the qPCR. This was used as input sample for the qPCR analysis. The same procedure was performed on the beads of the last of the three negative selection round to quantify the amount of peptides sticking to the beads (negative selection). These two samples were compared to the input library, *i.e.* before exposure to the beads, which was diluted 500 times in MQ before PCR amplification of which 1 μL was used in the qPCR mix. The input, positive and negative samples were amplified and compared to a standard curve prepared from a dilution series of reverse transcribed input mRNA library without translation. The process was repeated until the positive selection displayed superior enrichment compared to the negative selection. DNA output was then sequenced using a Illumina ISeq platform using a 2 × 150 bp V2 reagent kit at the VUMC Medical Genomics sequencing facility (Amsterdam, NL). Sequencing data was processed using python scripts to filter for the expected T7 promotor and puromycin linking sequences, converted from DNA to peptide sequence, and further filtered for peptides of correct length. The sequence alignments and phylogenetic tree generation were performed using CLC sequence viewer 8.0.

### Peptide synthesis

Peptide synthesis for the initial selection of the various RaPID family sequences was performed at 25 μM scale on a Syro Multisyntech Automated Peptide synthesizer (SYRO robot; Part Nr. Syro II; Serial: 2015-05-01 Syro II; Multisyntech GmbH, Germany, under inert gas (N_2_) application). The machine was washed with NMP and the resin was swelled with NMP for 5 minutes. Fmoc deprotections were performed using 20% piperidine in NMP. Coupling reactions were performed using PyBOP and DIPEA. After the last coupling steps the resin was washed manually using DCM and diethyl ether. The resin was left to dry. Coupling of chloroacetic acid and subsequent cyclization was performed as described in general procedure B, C and D in the work of van Haren *et al*.^27^ For the initial selection these were checked on LCMS and not purified further, data not shown. For all subsequent syntheses of cL3 variants procedures were identical, though at larger scale (0.5 mmol) and peptides were purified *via* reverse phase HPLC and freeze-dried. QC data and methods are described in the ESI.† The deletion scan products were ordered from GenScript (United Kingdom) at a purity of at least 75% after freeze-drying to a white powdered form. Peptide structures and analytical HPLC data can be found in the ESI† including analytical HPLC, amino acid sequences, structures and for the sequences and HRMS data. Analytical HPLC and HRMS procedures are described in the ESI.†

### Protein expression

The C1qNB75 plasmid was a gift from Nick Laursen.^25^ C1qNB75 was produced using BL21 DE3, which were grown to an OD600 of 0.6 in lysogeny broth. Samples were oxygenated by shaking at 200 rpm and kept at 37 °C. After the appropriate OD was reached, 0.5 mM of Isopropyl β-d-1-thiogalactopyranoside (IPTG) (VWR chemicals, NL) was added to induce protein synthesis and bacteria were incubated at 20 °C with shaking for approximately 16 hours. Bacteria were collected in a pellet by centrifugation. Cells were lysed in cold wash buffer (300 mM NaCl, 20 mM Tris–HCl, and 20 mM imidazole, pH 8) using probe sonication and debris removed at 24 000g for 40 minutes at 4 °C. HisPur^TM^ NiNTA (Thermofisher Scientific) beads were used to purify the protein. Columns were equilibrated with 10 column volumes of wash buffer after which the supernatant was added to the column. Wash buffer with increasing concentrations of imidazole was added to the column until a concentration of 250 mM imidazole was reached. The eluted protein fractions were collected and purified further using size exclusion chromatography (S200 Superdex® prep grade), which had been equilibrated with PBS. Samples were concentrated using Amicon® spin filters and stored at −80 °C. Alemtuzumab (antiCD52 IgG1) was a gift from the Trouw lab (LUMC, NL).

### Enzyme-linked immunosorbent assay (ELISA) for complement activation

Maxisorb Nunc Immunoplates (Thermofisher Scientific, Massachusetts, USA) were coated with streptavidin (Thermofisher Scientific) at 10 μg mL^−1^ in 0.1 M Na_2_CO_3_, 0.1 M NaHCO_3_ (coating buffer), pH 9.6 at 50 μL per well and allowed to incubate either at room temperature overnight, or 37 °C for 1 hour. Plates were washed with PBS with 0.05% tween-20 three times. For each following step, samples were incubated at 37 °C for 1 hour and subsequently washed with the PBS-T solution, we denote this as incubation and wash in the latter parts of the text. After streptavidin binding, wells were blocked with 100 μL of 0.1 M spermidine (Thermofisher Scientific) in distilled water incubated and washed. Then peptides were incubated in 50 μL PBS (as with all the following steps) with 0.05% Tween-20 (PBS-T) to create a peptide presenting surface and incubated and washed. In all activation assays, peptides are modified with a biotin to achieve binding to streptavidin. Note that in certain instances the peptides are initially modified with an azide. In such conditions a biotin-PEG4-DBCO (Jena Bioscience GmbH, Germany) was co-incubated with the peptides at 25 mol% excess of biotin linker to create a biotin modified peptide. This conjugation reaction took place in the plate. Hereafter the wells were incubated with 1% normal human serum (NHS) (Complement Technology, Inc., Texas, USA) in RPMI medium (Roswell Park Memorial Institute 1640 medium), then incubated and washed. Following these steps, complement components C1q or C5b-9 were detected. Primary Rabbit anti-C1q or Mouse anti C5b-9 (Dako, Denmark) was added to PBS, 0.05% Tween-20, 0.1% BSA (PBS-BT) in 1 : 2000 and 1 : 333 dilution, respectively, then incubated and washed. Next, goat anti-rabbit and anti-mouse antibodies (Dako, Denmark) modified with horseradish peroxidase (HRP) were diluted 1 : 5000 in PBS-T and added to the plates, then incubated and washed. To detect the HRP on the secondary antibody 2.5 mg mL^−1^ ABTS (2′-azino-bis(3-ethylbenzothiazoline-6-sulfonic acid)) was added in citric acid buffer (0.15 M, pH 4.2) (ABTS buffer) containing 0.15 (v/v%) H_2_O_2_ was used and samples were incubated at room temperature for 30 minutes. Absorption measurements at 415 nm were performed on a CLARIOstar microplate reader (BMG Labtech, Offenburg, Germany).

### Competition ELISAs

Materials, including buffers and blocking agent, were the same as for the activation assay, unless stated otherwise. Incubation and wash steps between added new components was kept identical to the section “*Enzyme-linked immunosorbent assay (ELISA) for complement activation”* above. To determine the competition with pooled human IgG (Sigma-Aldrich, USA), Maxisorb plates were coated with 10 μg mL^−1^ of IgG pooled from human serum, incubated, washed and blocked with spermidine. Peptides were incubated with 1% normal human serum (pooled, Complement Technology) for at least 30 minutes before adding to the IgG coated plates. DMSO controls and no serum controls were added. Human serum with and without peptides was added to the plates and subsequent complement inhibition was determined by detecting the presence of C1q or C5b-9. Data shown in the graphs is normalized to the maximum signal of the DMSO control and the minimal signal of the no serum control. Absorption measurements at 415 were performed on a CLARIOstar microplate reader (BMG Labtech, Offenburg, Germany).

To perform the competition assays to assess overlap in the binding region of gC1q to cL3 and either C1qNB75 or CRP, buffers, materials and sample volumes were as described above. C1qNB75 or CRP (Invitrogen) were added to the Maxisorb plates at 10 μg mL^−1^ in binding buffer and incubated at 37 °C for one hour, then washed three times with PBS-T. In all further steps plates were incubated at 37 °C and then washed each time. After coating the plates with the proteins, wells were blocked with spermidine. Then human serum, 1% in RPMI was added to the plates in presence or absence of 20 μM of cL3 for the antibodies. For CRP a titration of cL3 was performed. Subsequently complement components C1q and C5b-9 were detected and absorbance as a consequence of ABTS conversion was measured.

To immobilise cL3, wells were coated with streptavidin 10 μg mL^−1^ in coating buffer. Then wells were blocked with spermidine and subsequently 20 μM of cL3 was added to the plates, incubated and washed. Then human serum (1% in RPMI) was added to the wells in the presence or absence of 100 nM of C1qNB75. Complement components C1q of C5b-9 were detected and ABTS conversion measured.

### Liposome preparation

Liposomes were prepared using DMPC:DMPC:CHOL:DBCO (1,2-dimyristoyl-*sn*-glycero-3-phosphocholine:1,2-dimyristoyl-*sn*-glycero-3-phospho-(1′-rac-glycerol) (sodium salt):Cholesterol (ovine): 1,2-dipalmitoyl-*sn*-glycero-3-phosphoethanolamine-*N*-dibenzocyclooctyl) 44 : 5 : 50 : 1 molar%. Liposomes were ordered from Avanti Polar lipids (Pelham, USA). The lipids were dissolved in glass vials in a chloroform/methanol mixture 9 : 1 v/v mix. Lipids were then added to 1 mg of total lipid mass and the organic solvent mix was evaporated using N_2_ for at least 2 hours. Aqueous buffer, 1.2 mL was used to rehydrate the lipids, the buffer composition is specified in the relevant sections below. The vials were incubated in a water bath at 50–60 °C for one hour and agitated by pipetting to resuspend. Liposomes were extruded through with a 400 nm polycarbonate membrane using a mini-extruder (Avanti Polar Lipids). These liposomes were stored at 4 °C until further use for up to two weeks.

Conjugation of the peptides onto the liposomes surfaces was achieved using copper-free click chemistry between the azide handle on the peptides and the DBCO-coated liposomes by incubating overnight. The peptide was added at 20× excess to the DBCO lipid handle. Unreacted peptide was removed from the sample by centrifugation at 20 000 g for 15 minutes at 4 °C, supernatant was removed and liposomes resuspended in the relevant assay buffer. In the assays the blank liposome controls constitute liposomes that contain unreacted DBCO from the same liposome batch.

### C1s activity assay

Using either peptide modified liposomes or blank liposomes, C1s enzymatic activity of was monitored using a substrate conversion assay. C1s substrate Boc-Leu-Gly-Arg-AMC (Amino Methyl Coumarin) – PeptaNova GmbH (Sandhausen, Germany) (LGR-AMC) was incubated with purified C1 protein (Complement Technology, Texas, USA). Pure C1 was buffer exchanged using 100 kDa spin filters (Amicon) into 150 mM NaCl 50 mM Tris-HCl pH 7.5, 5 mM CaCl_2_ (assay buffer). After all components were mixed, the substrate was added at 500 μM, diluted from a 10 mM stock (5% DMSO final concentration). C1 protein concentration was kept at 40 nM for all these experiments. It is important to note that when using purified C1 protein (from Complement Technology), the sample has to be buffer exchanged from the manufacturer storage solution. Liposomes were formed in PBS and, after conjugation of the peptide using copper-free click chemistry, buffer exchanged to assay buffer *via* centrifugation as described above. Fluorescent signal over time from the AMC was monitored on a CLARIOstar microplate reader (BMG Labtech, Offenburg, Germany). Measurements were taken every minute for a period of 5 hours with excitation and emission set at 360 and 460 nm, respectively.

### Liposome lysis assay

The liposome lysis assay monitors the formation of the membrane attack complex upon complement activation. Peptide-modified liposomes were exposed to 10% human serum (Complement Technology) in PBS and then peptides recruit C1 to the membrane leading to activation of C1 and the downstream complement pathway. Liposomes were prepared to contain 20 mM sulforhodamine B (SRB) (Sigma Aldrich, St louis, MO, USA), in PBS. During formation liposomes are prepared in PBS buffer containing 20 mM SRB and subsequently excess SRB is removed using NAP-25 columns (Cytiva NAP^TM^). The content release of SRB is monitored over time (every 15 seconds) on a CLARIOstar microplate reader (BMG Labtech, Offenburg, Germany).

### Complement inhibition assay

A cell mimetic system was used as in the liposome lysis assay. Liposomes encapsulating SRB (20 mM in PBS) were synthesised displaying 1 mol% cholesterol-modified CD52 mimotope.^2^ Liposomes were pre-incubated with 500 nM of anti-CD52 IgG1 (alemtuzumab). Before adding 10% human serum to the liposomes, various concentrations of peptide were added to the serum and incubated for 10 minutes to 1 hour. For the titrations samples were incubated for 10 minutes. Buffer and DMSO controls were added and control sample with no antibody was added to act as baseline signal. Data was collected as in the liposome lysis assay. Statistical analysis for the complement inhibition assay was performed using GraphPad Prism (version 9).

## Author contributions

SMWRH performed all experimental work and data analysis. LA collected preliminary data. SMWRH and SAKJ performed RaPID selection and sequence analysis. ALB and THS conceived the project. SMWRH and THS interpreted the data and wrote the paper. All authors contributed to and approved the manuscript.

## Data availability

The high throughput sequencing data of the library from the RaPID selection are available in the DataverseNL repository at this https://doi.org/10.34894/CXWYPT.^60^

## Conflicts of interest

SMWRH, LA, ALB and THS are co-authors on a patent describing the development and use of peptactins. SAKJ declares no competing interests.

## Supplementary Material

CB-005-D4CB00081A-s001
