# Supplementary material for: Selection and characterization of a peptide-based complement modulator targeting C1 of the innate immune system
Source: RSC Chem Biol. 2024 Jul 1;5(8):787–99. doi: 10.1039/d4cb00081a (PMC11289891; doi:10.1039/d4cb00081a)
Supplement: CB-005-D4CB00081A-s001 [file CB-005-D4CB00081A-s001.pdf]

Electronic Supplementary Material (ESI) for RSC Chemical Biology.
This journal is © The Royal Society of Chemistry 2024

SI

Selection and characterization of a peptide-based complement modulator targeting C1 of the innate immune system

Sebastiaan M.W.R. Hamers[1], Leoni Abendstein[1], Aimee L. Boyle[2,3], Seino A.K. Jongkees[4] & Thomas H. Sharp[1,5,*]

[1]Department of Cell and Chemical Biology, Leiden University Medical Centre, 2300 RC Leiden, The Netherlands
[2]Leiden Institute of Chemistry, Leiden University, 2333 CC Leiden, The Netherlands
[3]School of Chemistry, University of Bristol, Bristol, BS8 1QU, UK
[4]Department of Chemistry and Pharmaceutical Sciences, Vrije Universiteit Amsterdam, Amsterdam, The Netherlands
[5]School of Biochemistry, University of Bristol, Bristol, BS8 1TD, UK

*To whom correspondence should be addressed: t.sharp@bristol.ac.uk

Keywords:
Complement, RaPID, peptide selection, agonistic therapeutic, structure-guided drug design

SI methods:

QC of the peptides

Analytical HPLC
cL3-Az, cL3-Bt, cL3-inv and cL3-lin were analyzed on a Shimadzu Prominence-I LC2030 system equipped with a Dr Maish ReproSil Gold 120 C18 column (4.6 * 250 mm, 5 µm). Samples were run at 30 °C and UV detection was performed at 214 nm. These flow systems were used: buffer A 95/5 vol% water (ultrapure)/ACN with 0.1% TFA and buffer B 5/95 water/ACN 0.1% TFA. The gradient was set at 0% buffer B for 2 min, then B concentration was ramped to 100% over the course of 25 min and kept there for 2 min. Buffer B concentration was then returned to 0 over the course of a minute and set at 0 for 3 more min. cL3 deletion variants were collected by Genscript. The solvent system was; Solvent A 0.065% TFA in water and solvent B 0.05% TFA in 100% ACN. Generally these were progressed from either a 5 vol% B to 65 or 35 in 25 min and then to 95% solvent B for 5 more min before going back to 5 vol% for 5 min. An Inertsil ODS-SP 4.6 x 250 mm column was used.

HRMS analysis
HRPMS analysis of the peptides was performed using a Shimadzu Nexera X2 UHPLC system equipped with a Waters Acquity HSS C18 column (2.1*100mm, 1.8 µm) and a diode array detector. Samples were measured at 30 °C using the following solvent system: Solvent A, 0.1% formic acid in water; solvent B 0.1% formic acid in acetonitrile. Flow rate was set at 0.5 mL/min. Gradient started at 95:5 A:B for one minute, the ramping to 15:85 for 6 minutes and 0.:100 over the course of a minute and kept there for 3 minutes. Mass detection was performed in a Shimadzu 9030 QTOF mass spectrometer that had been calibrated with Agilent's API-TOF reference mass solution kit (5.0 mM purine, 100 mM ammonium trifluoroacetate and 2.5 mM hexakis (1H, 1H, 3H – tetrefluoropropoxy)-phosphazine). These were diluted to obtain a mass count of 10,000. Theoretical masses were calculated using Chemdraw 22.2.0 32-bit.

Figures S7-20: Analytical HPLC analysis of the peptides

HRMS data can be found tabulated in **Table S3** and sequences of the peptides in **Table S2**.

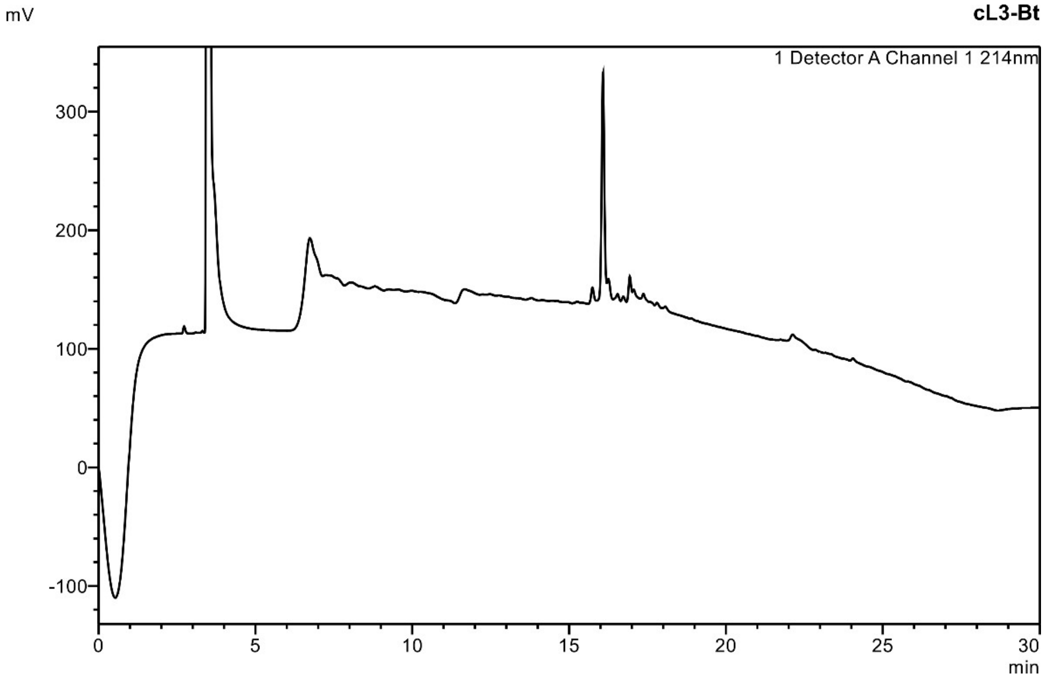
mV
cL3-Bt
1 Detector A Channel 1 214nm
300
200
100
0
-100
0
5
10
15
20
25
30
min

Figure S1

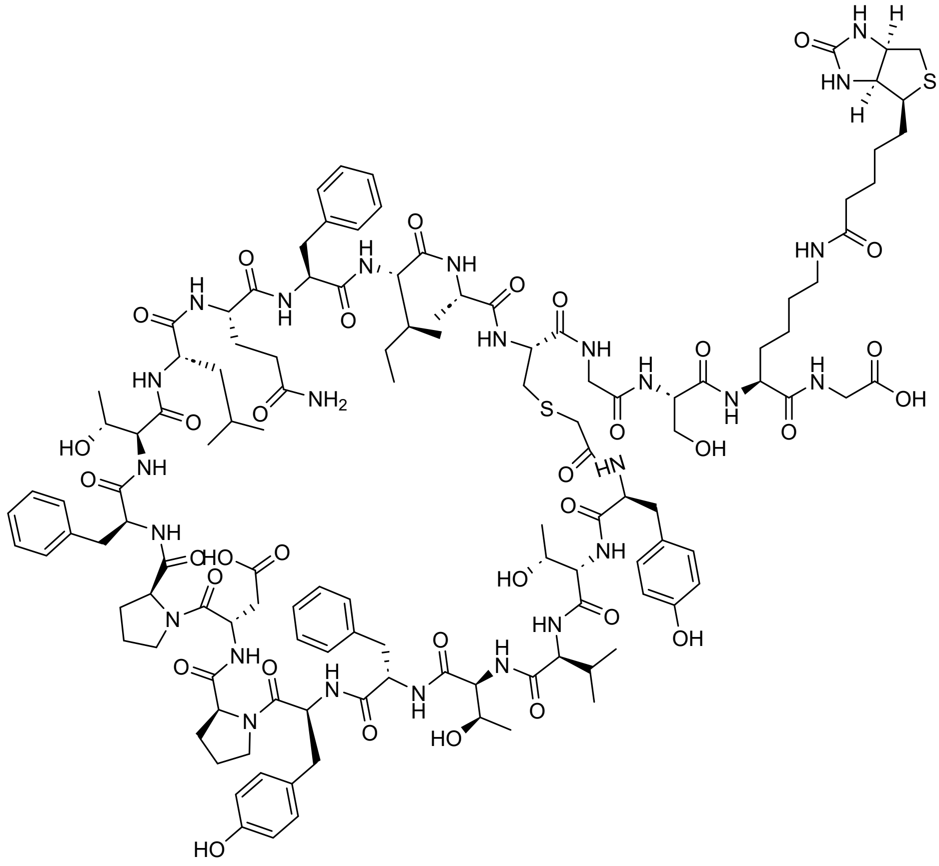

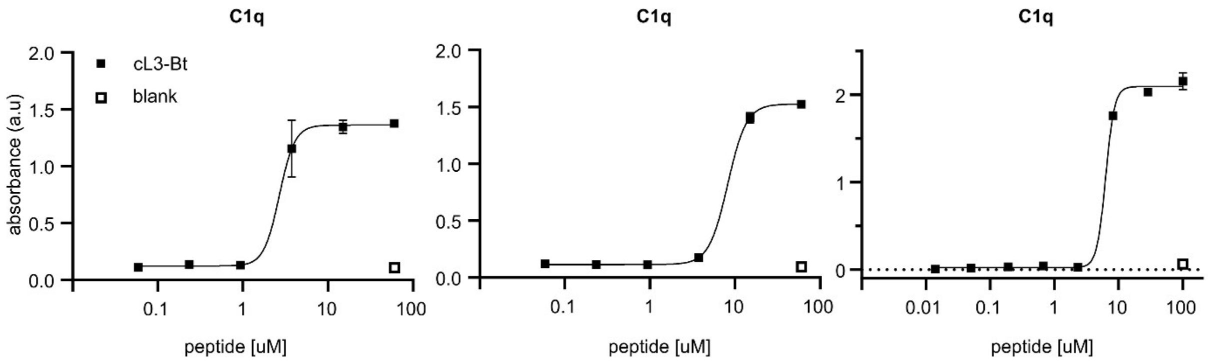

Figure S2: *ELISA detecting C1q recruitment from human serum (1% in RPMI medium) using cL3-Bt. cL3-Bt was titrated and allowed to bind streptavidin coated plates, herafter samples were washed to remove any unbound cL3-Bt and subsequently human serum was incubated on the plate. Each graph displays a technical duplicate collected on a different plate. cL3-Bt concentrations were slightly varied between plates. The datapoints on the right are also shown in* ***fig 3a****. Datapoints are shown in black squares and the line represents the fitted model. Graphpad Prism version 9.3.1 was used for model fitting using a non-linear dose vs response curve with four parameters least squares fit. Graph on the left: EC50: 2.68 , CI confidence interval (%)EC50: not determined (ND), R^2: 0.98. Graph in the middle: EC50:8.2 , CI confidence interval (%)EC50: 7.4-9.1 , R^2: 0.99. Graph on the right: EC50: 6.2 , CI confidence interval (%)EC50: ND, R^2: 0.99. Hence the EC50 as reported in the manuscript of ~6 µM*

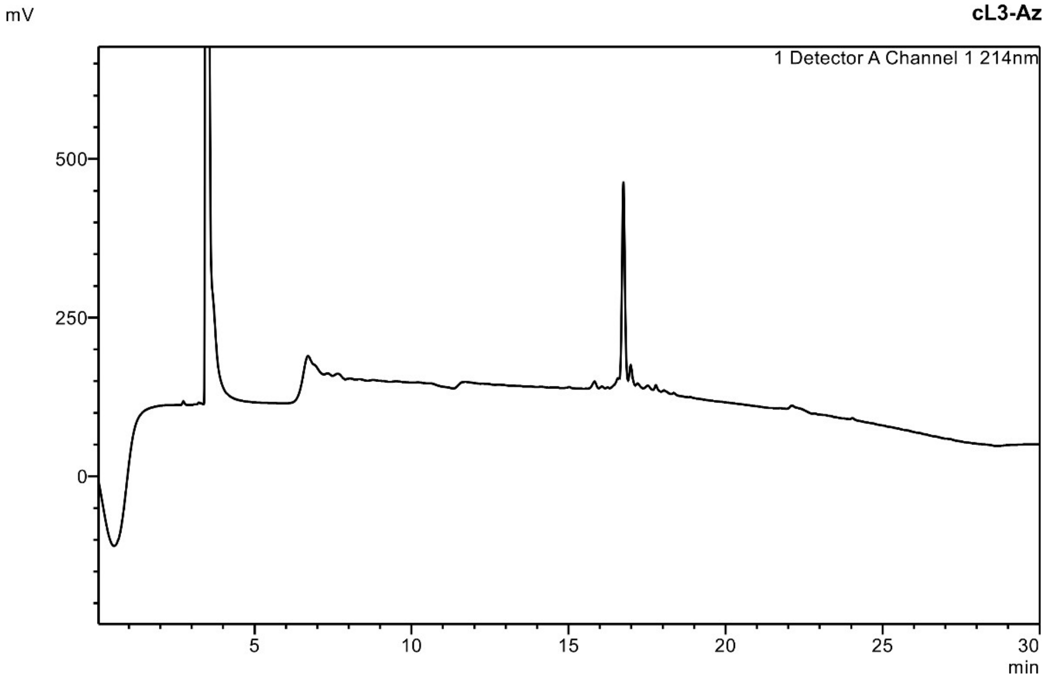
mV
cL3-Az
1 Detector A Channel 1 214nm
500
250
0
5
10
15
20
25
30
min

Figure S3

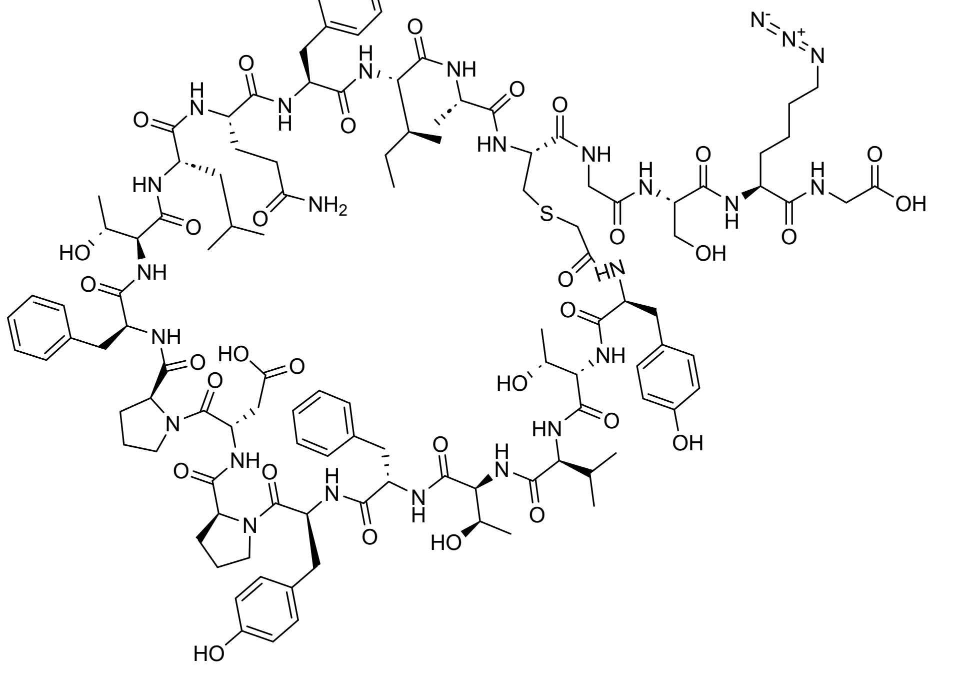

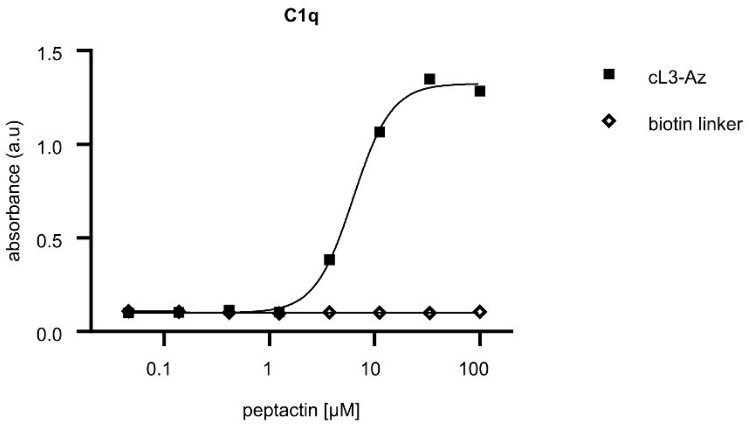

Figure S4: *ELISA of cL3-Az conjugated to a biotin-DCBO linker. Linker and cL3-Az were co-incubated on a streptavidin coated plate for 1 hour at 37 °C allowing for conjugation to take place. Hereafter unbound linker and peptide were washed off and human serum (1%, in RPMI medium) was added and subsequently C1q was detected. A single technical replicate is shown here. Datapoints are displayed in the black squares and the fitted model is the black line.* Graphpad *Prism version 9.3.1 was used for model fitting using a non-linear dose vs response curve with four parameters least squares fit. EC50:6.2, CI confidence interval (%) EC50: 5.2-7.4 ,* R^2*: 0.99.*

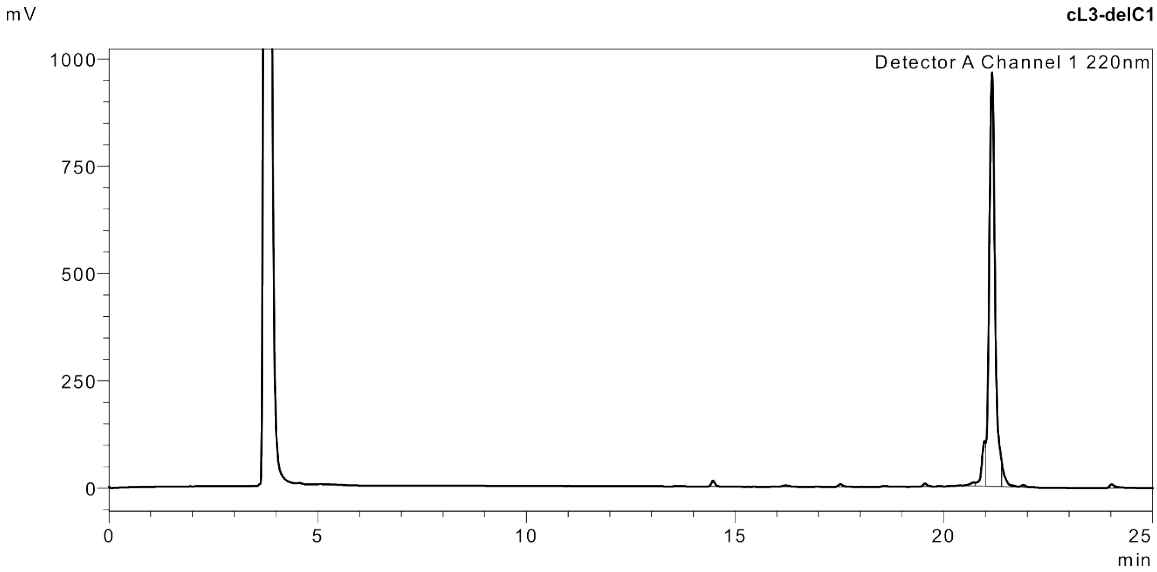
mV
cL3-delC1
Detector A Channel 1 220nm
1000
750
500
250
0
0
5
10
15
20
25
min

Figure S5

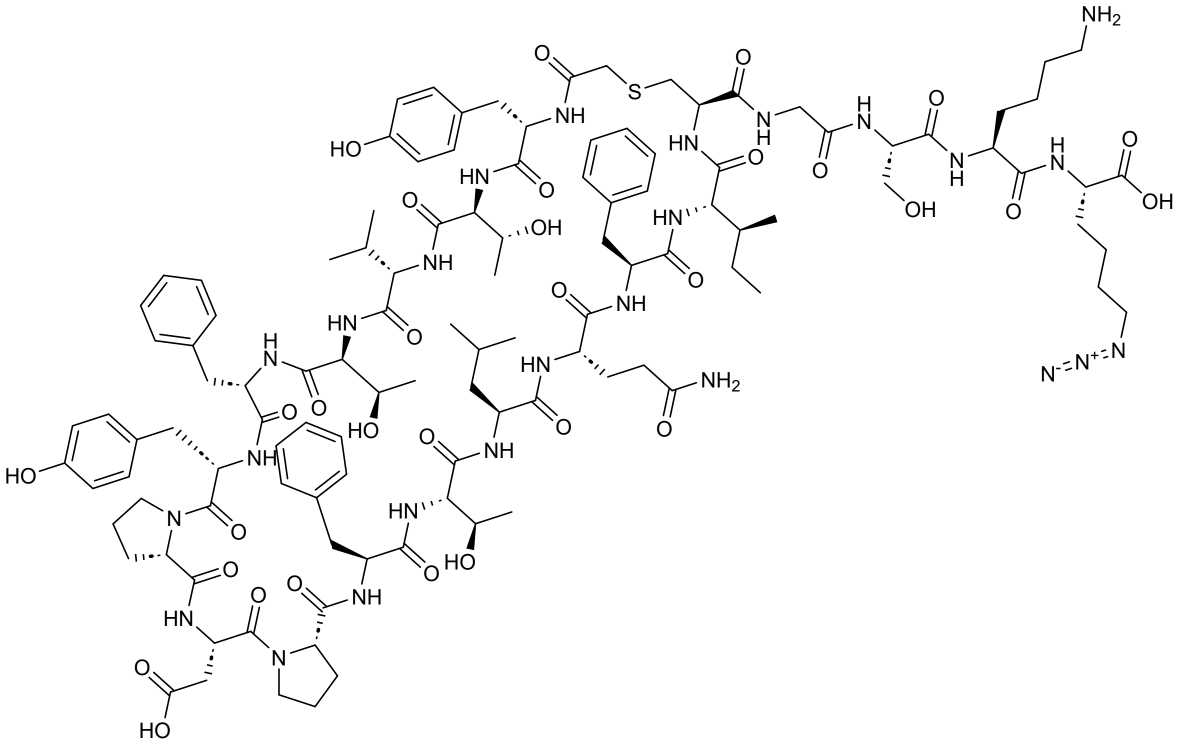

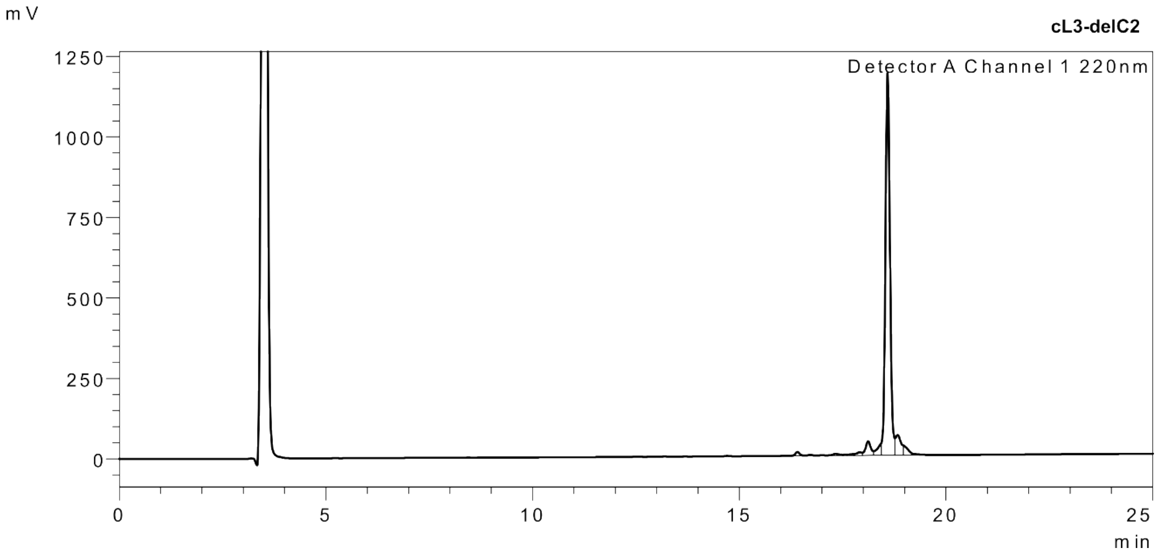
mV
cL3-delC2
Detector A Channel 1 220nm
1250
1000
750
500
250
0
0
5
10
15
20
25
min

Figure S6

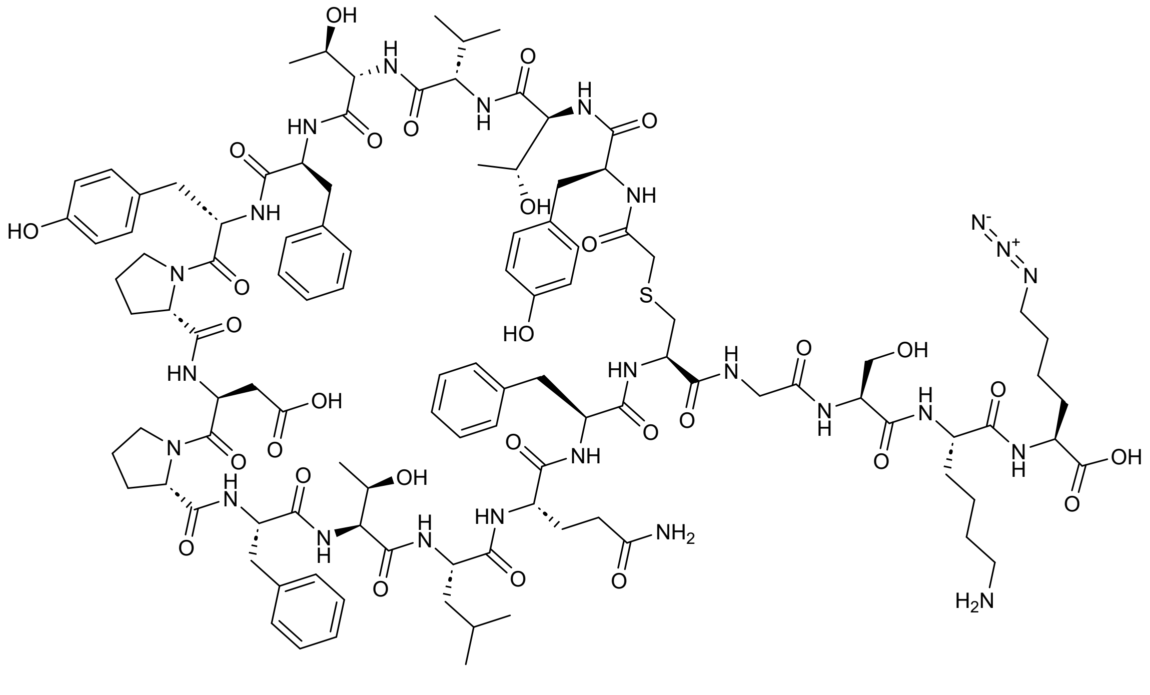

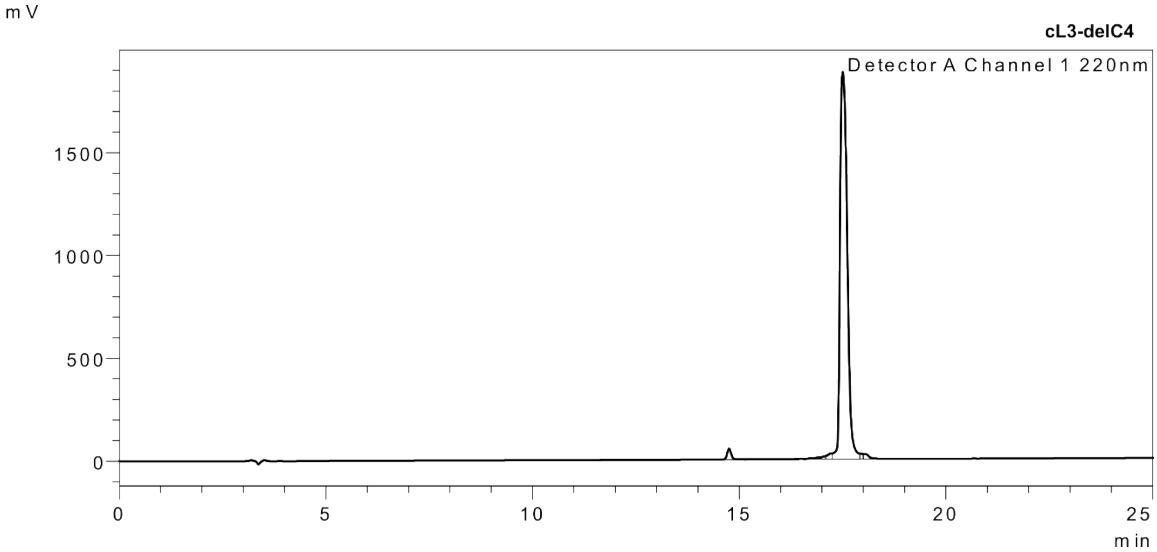
mV
cL3-delC4
Detector A Channel 1 220nm
1500
1000
500
0
0
5
10
15
20
25
min

Figure S7

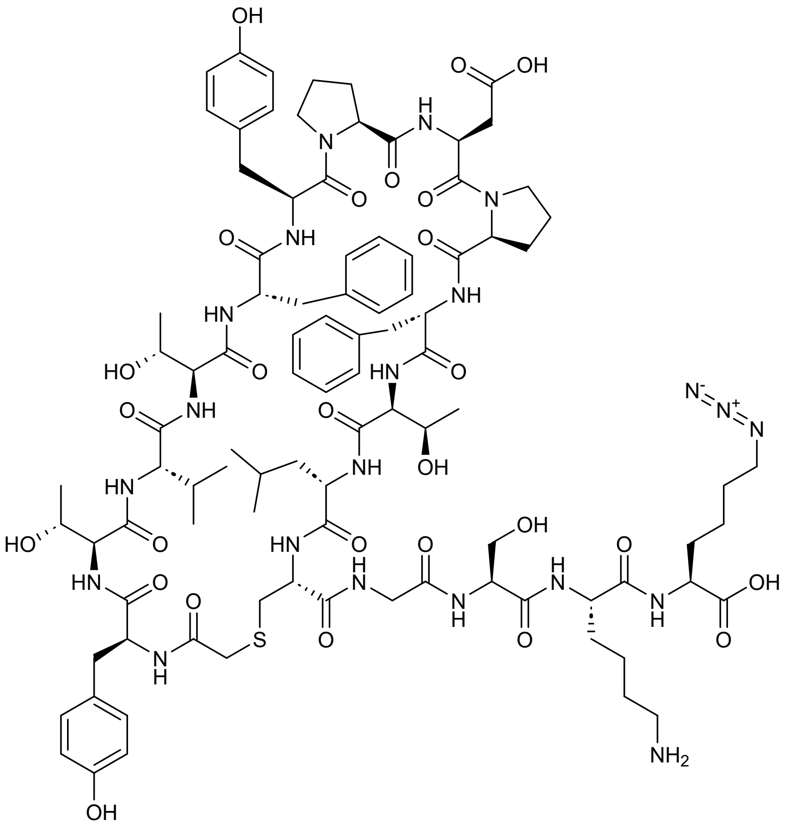

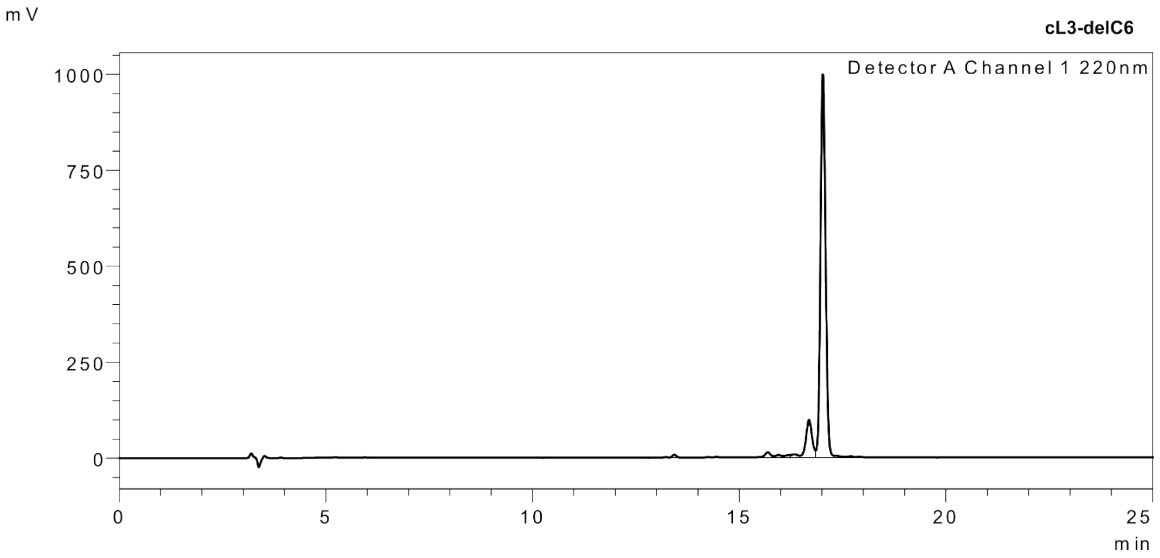
mV
cL3-delC6
Detector A Channel 1 220nm
1000
750
500
250
0
0
5
10
15
20
25
min

Figure S8

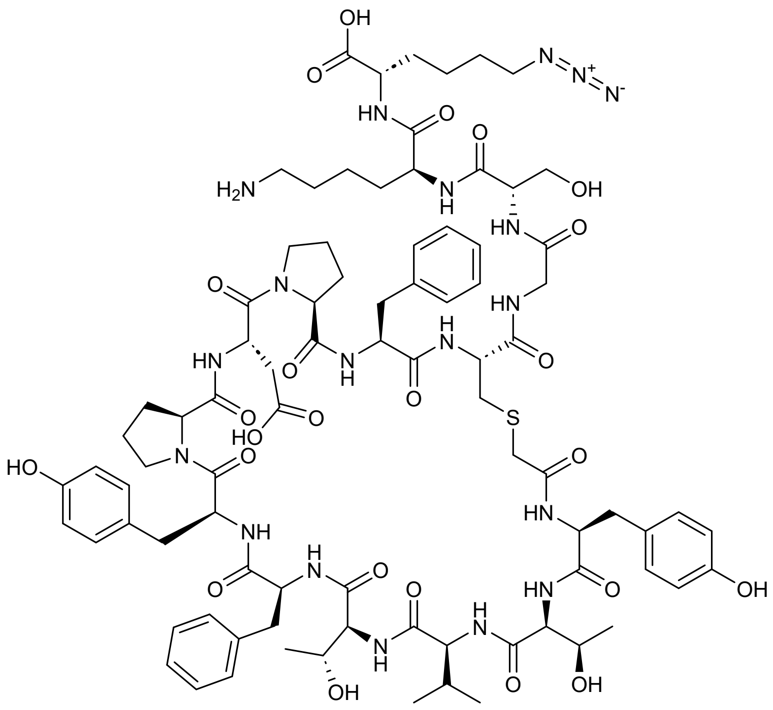
OH
O
N
N+
N-
HN
O
O
H2N
N
H
OH
HN
O
O
N
HN
H
N
HN
O
O
N
H
O
O
S
HO
O
O
HO
N
O
NH
HN
O
H
N
O
O
HN
O
OH
O
N
H
H
N
O
OH
OH

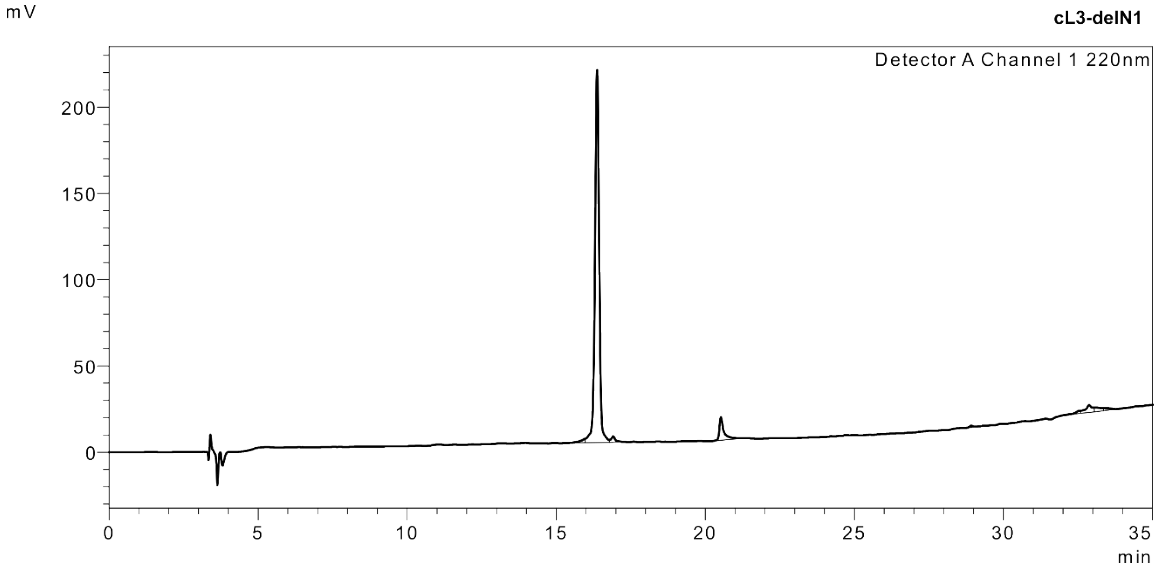
mV
cL3-delN1
Detector A Channel 1 220nm
200
150
100
50
0
0
5
10
15
20
25
30
35
min

Figure S9

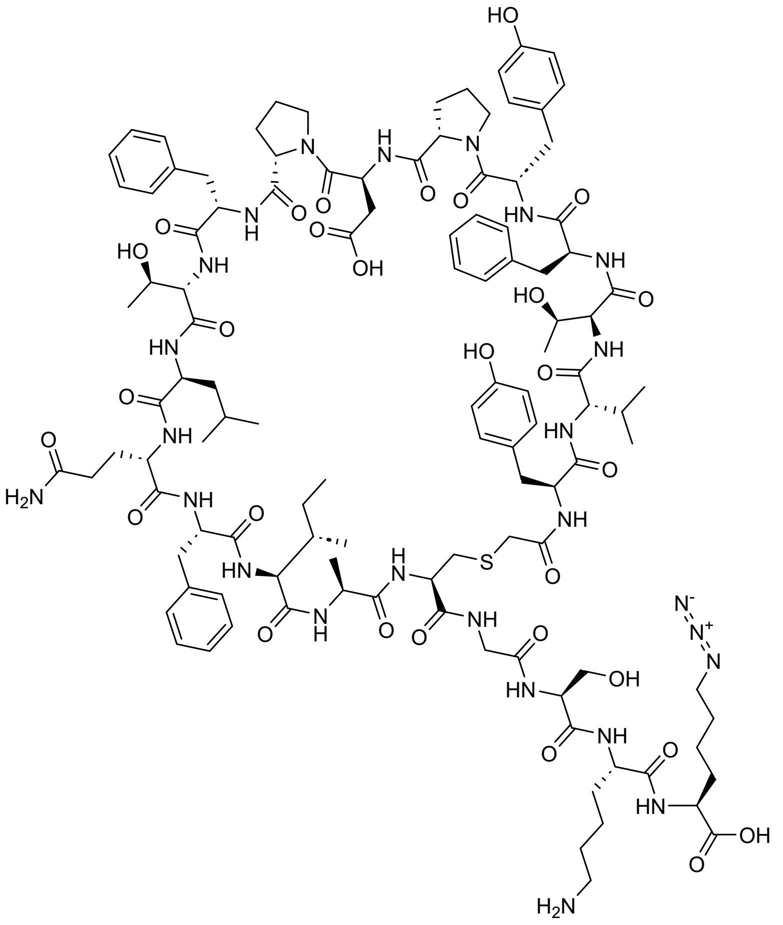

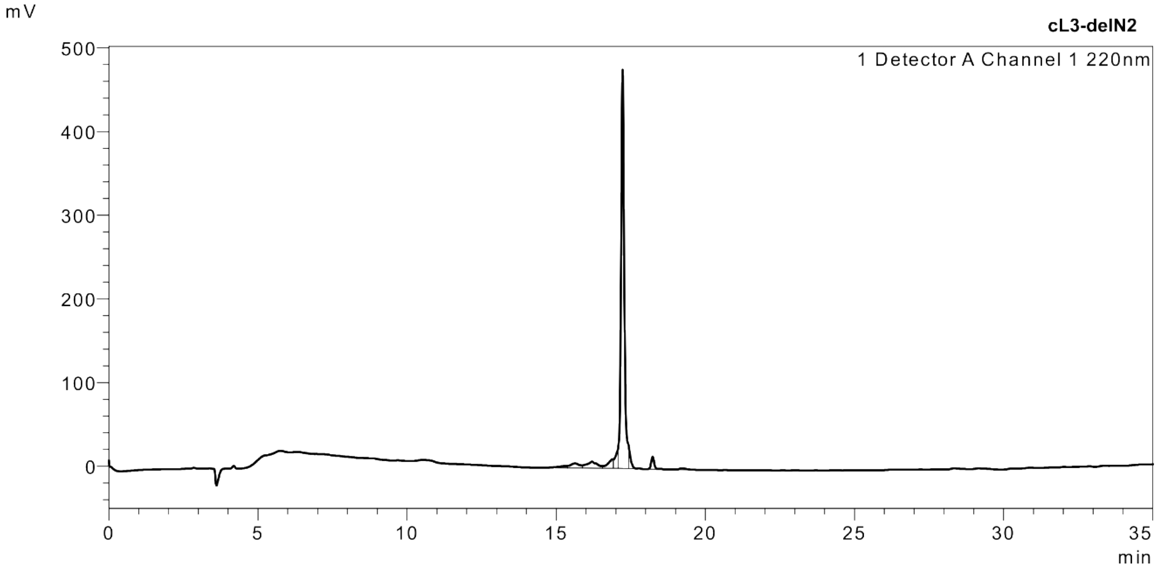
mV
cL3-delN2
1 Detector A Channel 1 220nm
500
400
300
200
100
0
0
5
10
15
20
25
30
35
min

Figure S10

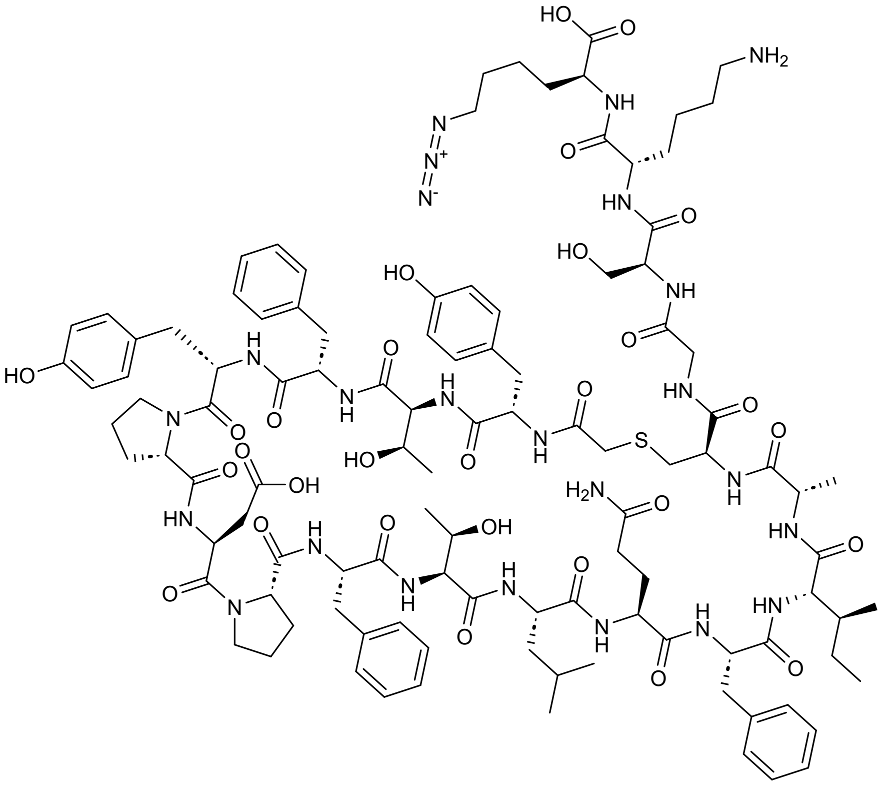

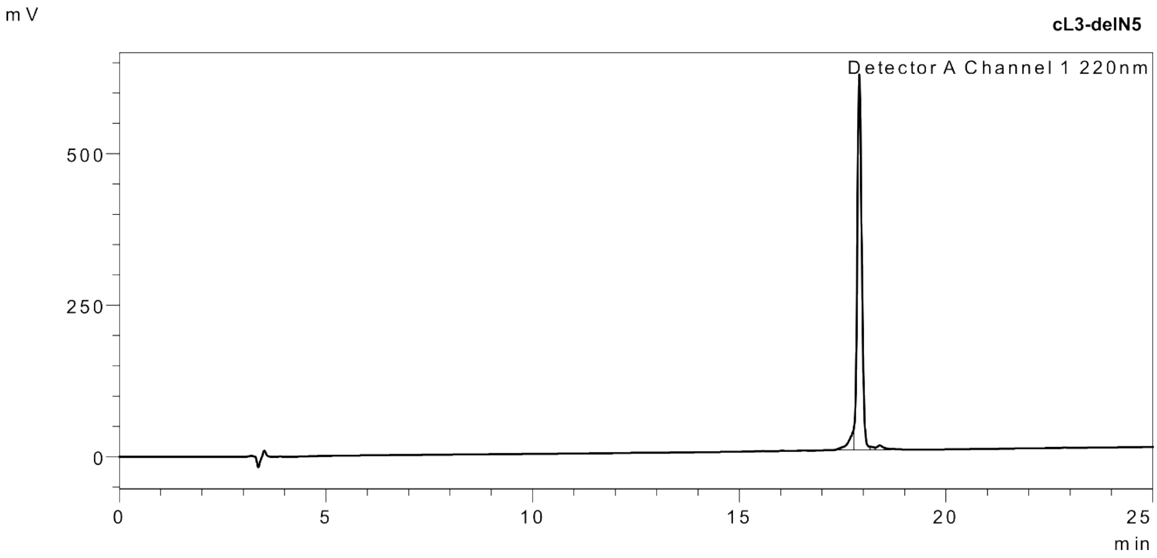

m V
cL3-delN5
Detector A Channel 1 220nm
500
250
0
0
5
10
15
20
25
m in

Figure S11

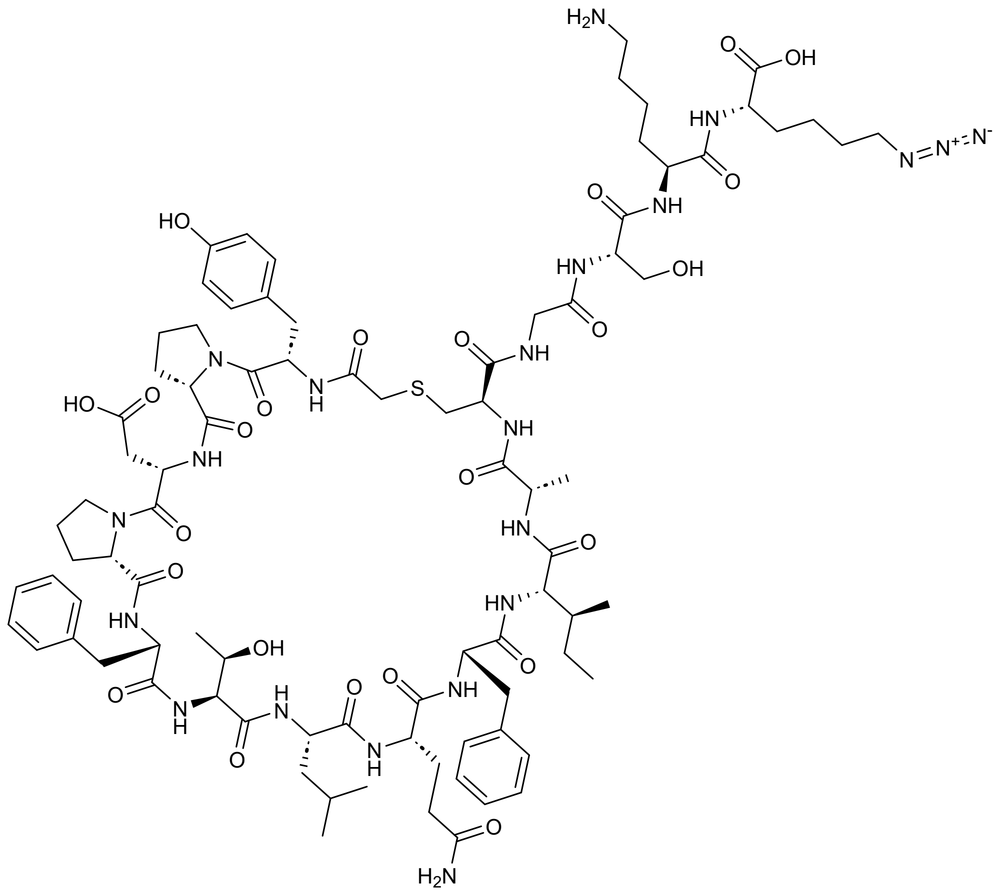

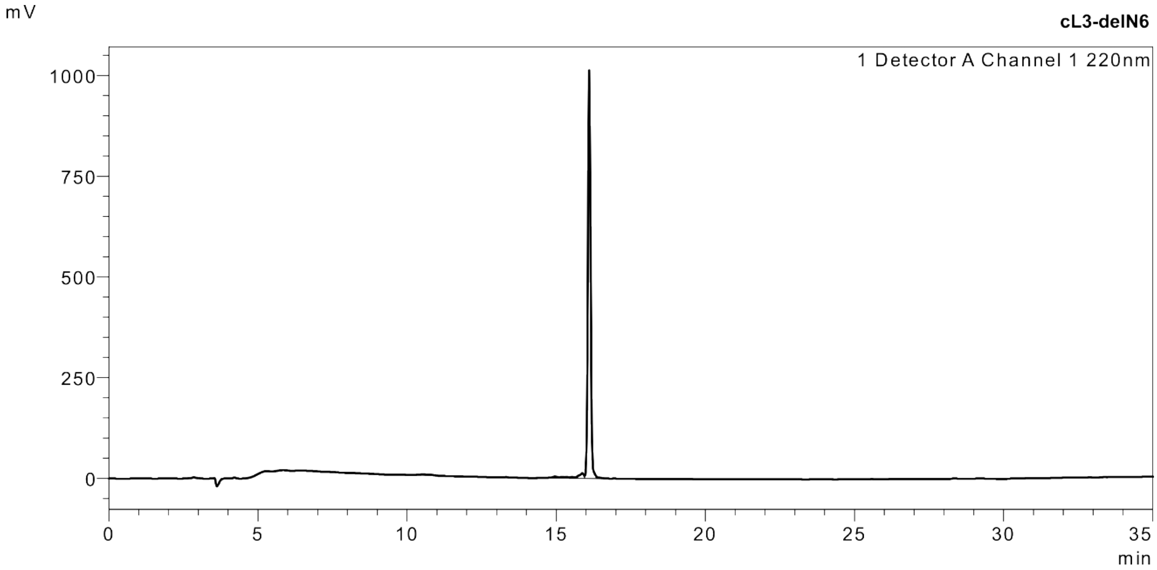
mV
cL3-delN6
1 Detector A Channel 1 220nm
1000
750
500
250
0
0
5
10
15
20
25
30
35
min

Figure S12

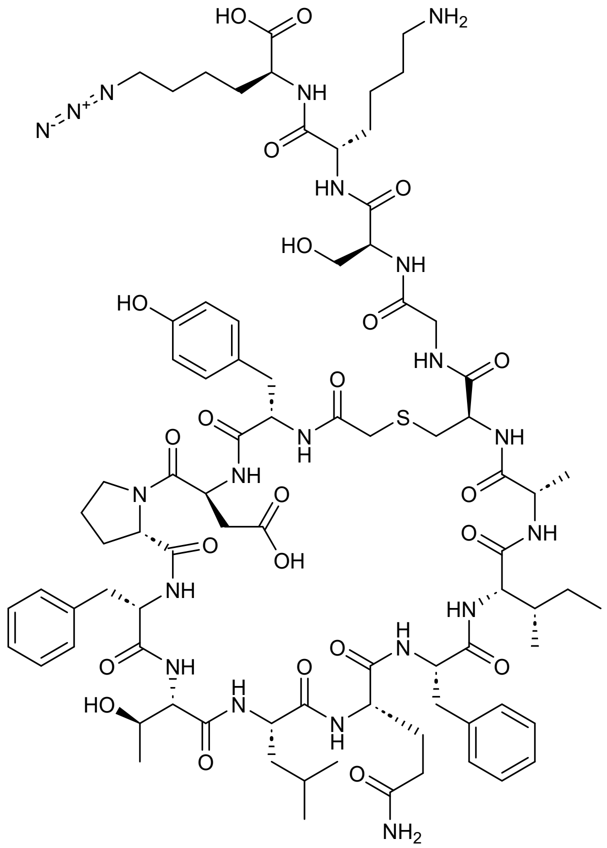
HO
O
NH2
N
N+
N-
NH
O
HN
O
HO
NH
HO
O
HN
O
O
O
N
H
S
NH
O
NH
O
N
O
NH
O
OH
O
NH
HN
H
N
O
O
NH
H
N
O
O
HO
O
N
H
O
NH2

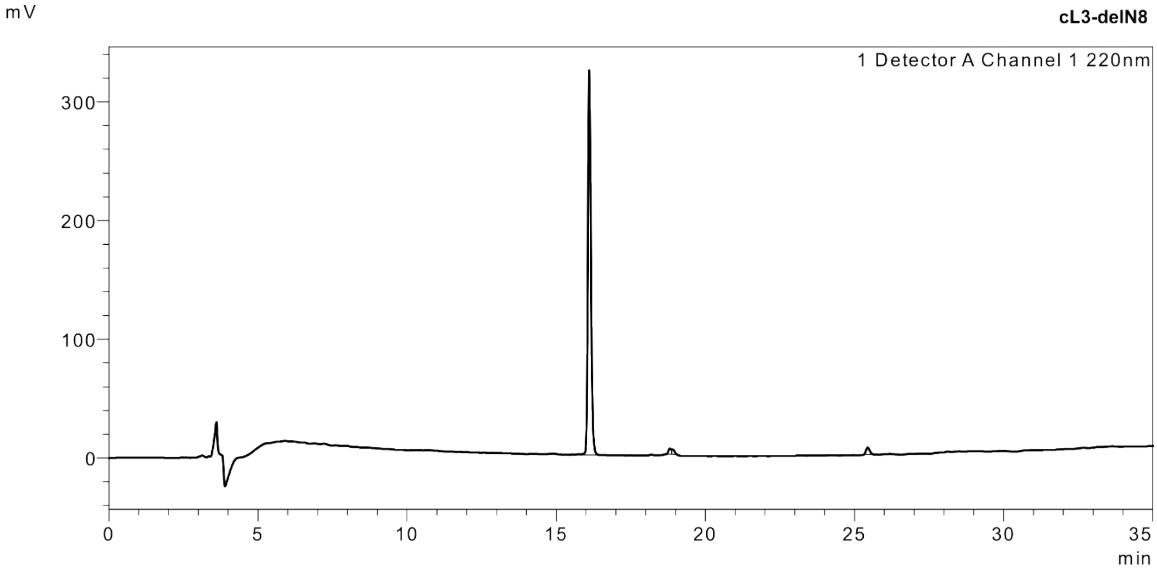
mV
cL3-delN8
1 Detector A Channel 1 220nm
300
200
100
0
0
5
10
15
20
25
30
35
min

Figure S13

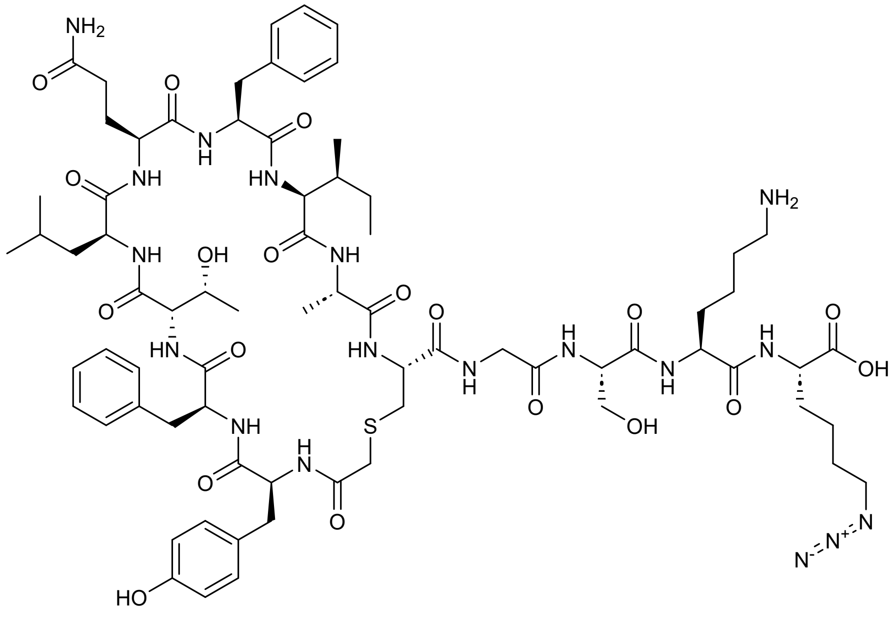
NH2
OH
HO
S
NH2
OH
OH
N-=N+=N

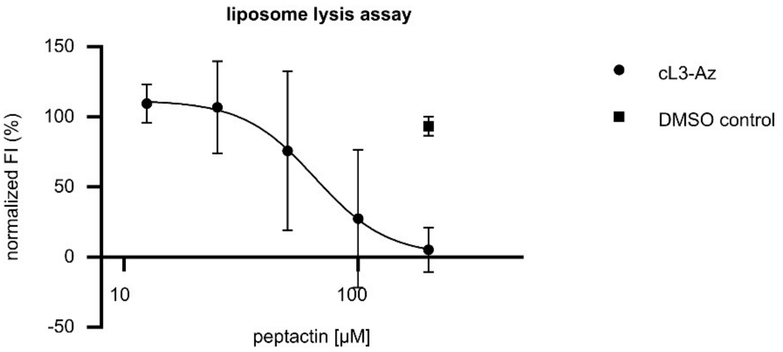

Figure S14: *Normalized FI data of the inhibition experiment displayed in **fig 5b**. Datapoints are normalized to the average values of the conditions where no Ab was present (min) and only Ab was present (max). Data was normalized using (FI-min)/(max-min)*100%. The DMSO control is to check for activity in the absense of cL3-Az. Datapoints are displayed and the fitted model is the black line.* Graphpad *Prism version 9.3.1 was used for model fitting using a non-linear dose vs response curve with four parameters least squares fit. IC50: 65 , CI confidence interval (%) IC50: ND, R^2: 0.62.*

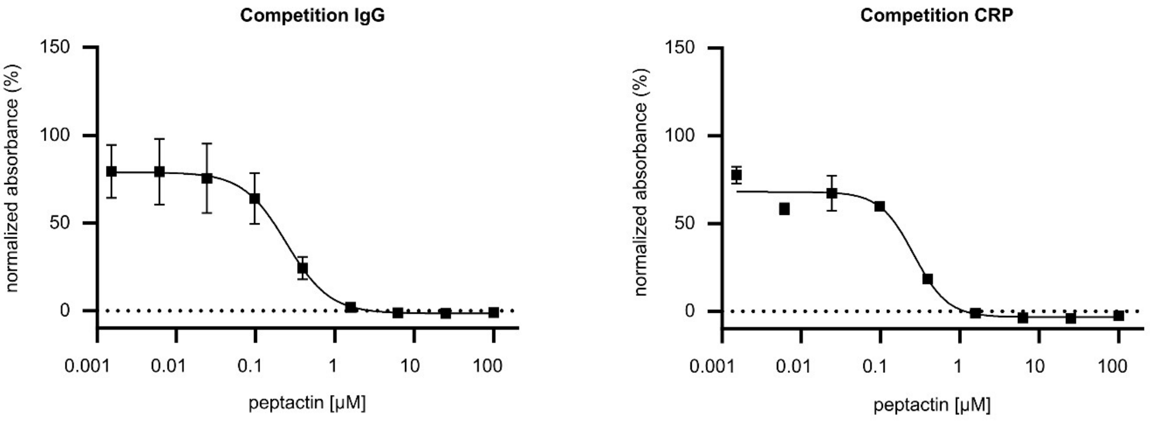

Figure S15: *Normalized absorbance data of the ELISA described in* ***fig 5c*** *(competition IgG) and* ***fig 7b*** *(Competition CRP). The datapoints are displayed and the line is the fitted model (technical duplicates for both). Datapoints are displayed and the fitted model is the black line.* Graphpad *Prism version 9.3.1 was used for model fitting using a non-linear dose vs response curve with four parameters least squares fit. Competition with IgG: IC50: 0.24 , CI confidence interval (%) IC50: 0.14-0.39 ,* R^2*: 0.94. Competition with CRP: IC50: 0.26 , CI confidence interval (%) IC50: ND,* R^2*: 0.97*

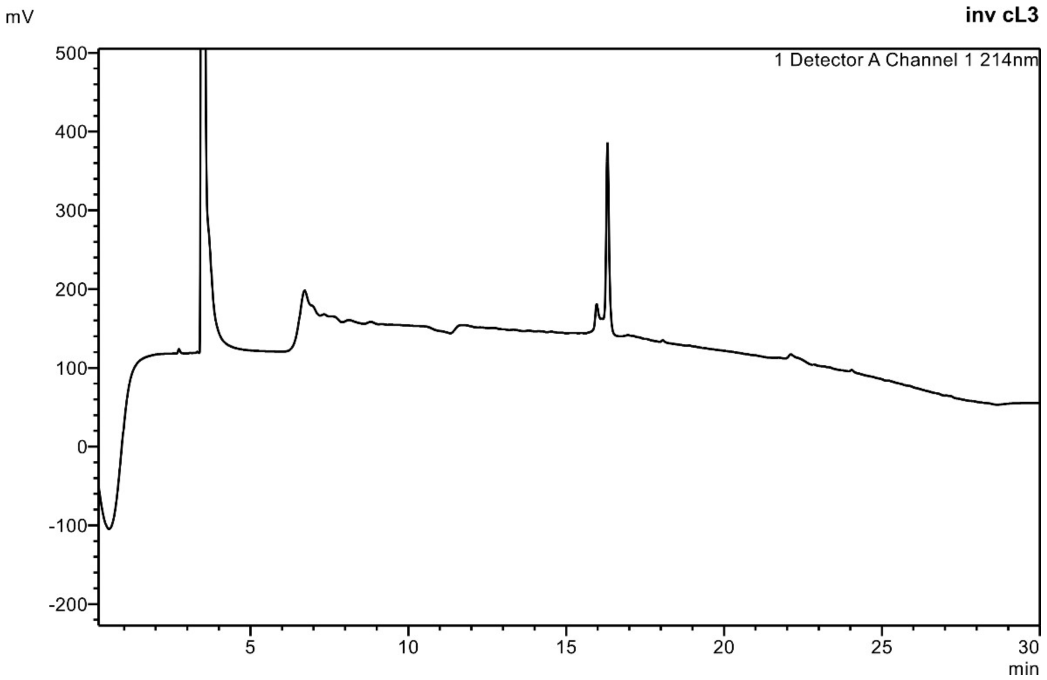
mV
inv cL3
1 Detector A Channel 1 214nm
500
400
300
200
100
0
-100
-200
5
10
15
20
25
30
min

Figure S16

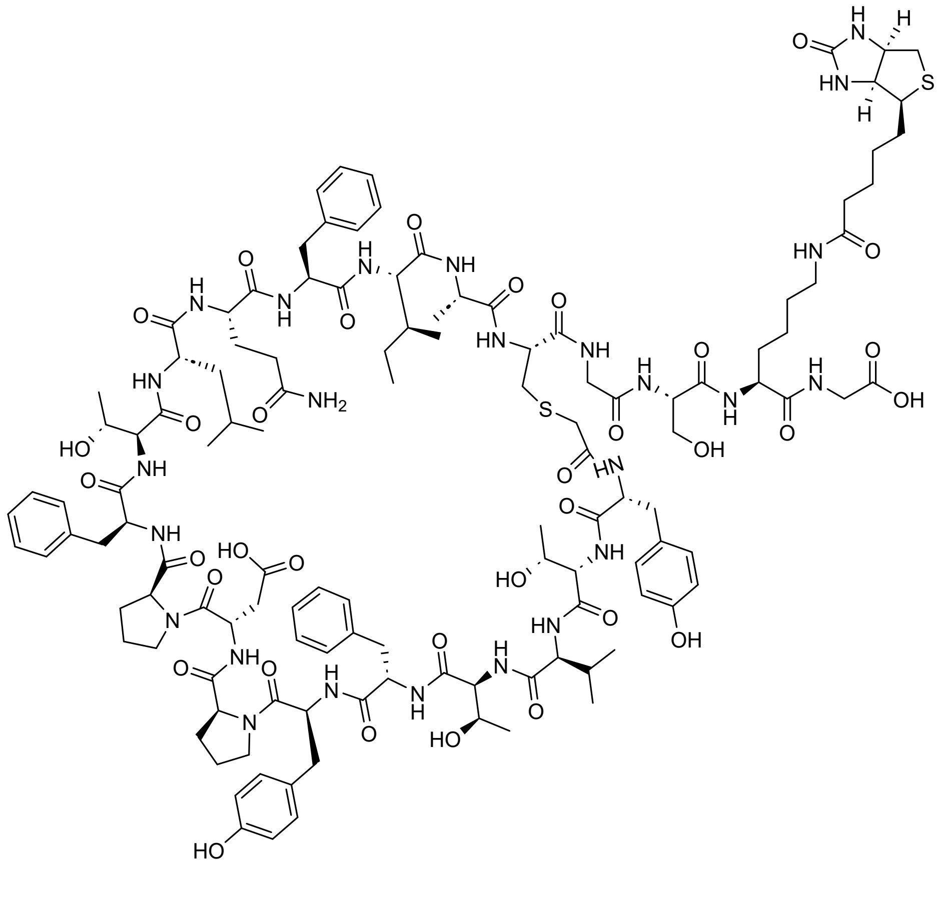

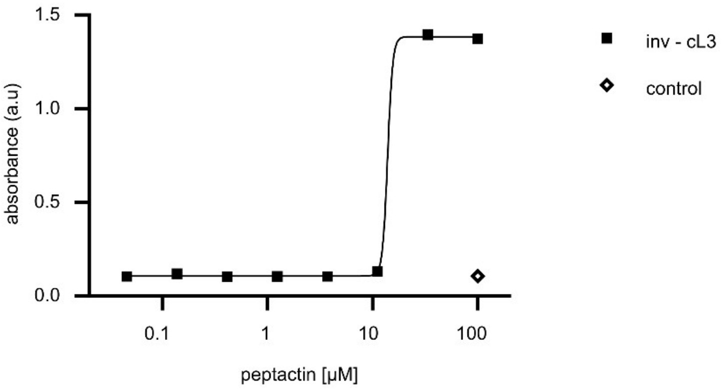

Figure S17: *absorbance data as described in **fig 6a**. Datapoints are shown and was fitted (black line) using Graphpad Prism version 9.3.1 for model fitting using a non-linear dose vs response curve with four parameters least squares fit. EC50: 13.9, CI confidence interval (%) EC50: ND,* R^2*: 0.99.*

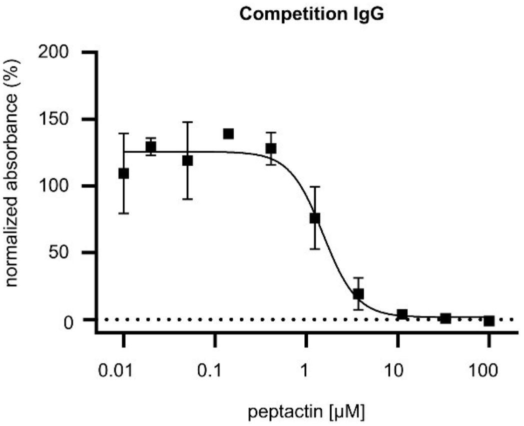

Figure S18: Normalized a*bsorbance data as described in **fig 6b**. Datapoints are shown and was fitted (black line) using Prism version 9.3.1 for model fitting using a non-linear dose vs response curve with four parameters least squares fit. Competition with IgG: IC50: 1.5, CI confidence interval (%) IC50: 1.01-2.13 , R^2: 0.94.*

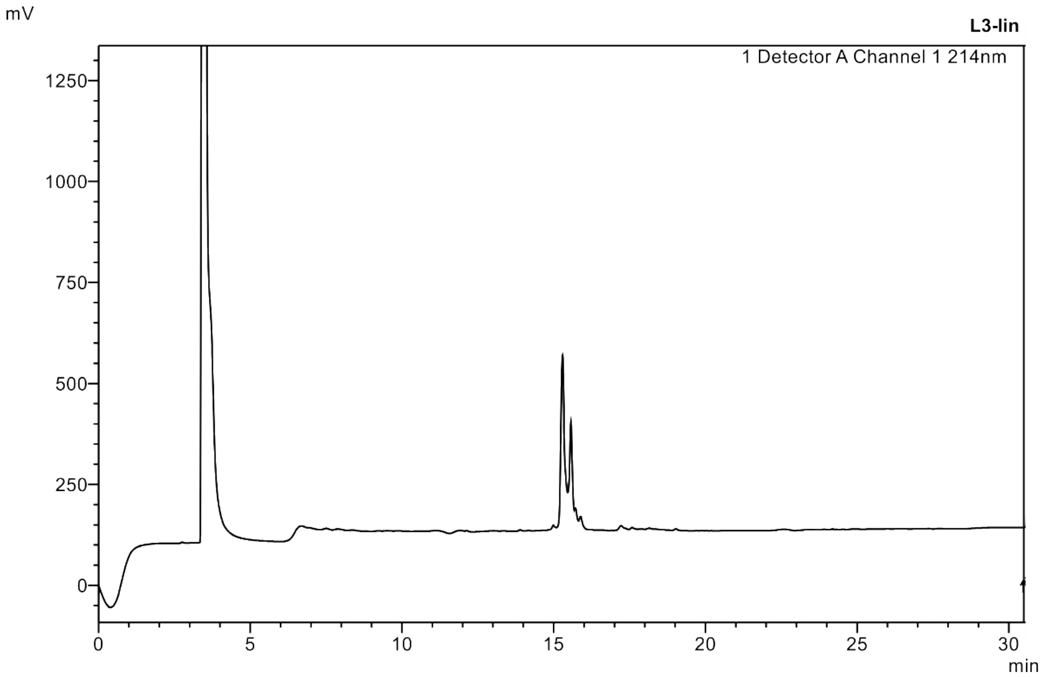
mV
L3-lin
1 Detector A Channel 1 214nm
1250
1000
750
500
250
0
0
5
10
15
20
25
30
min

Figure S19

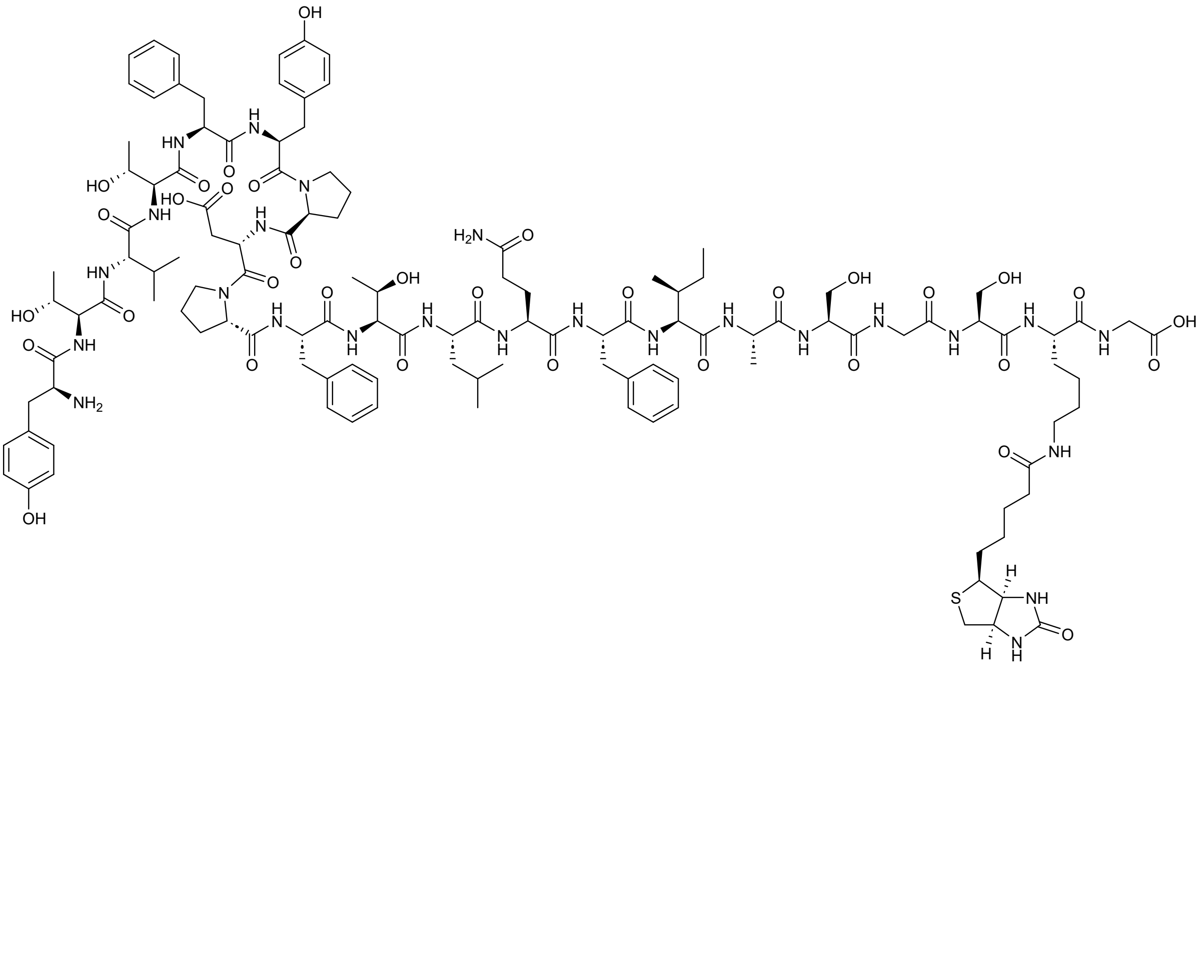

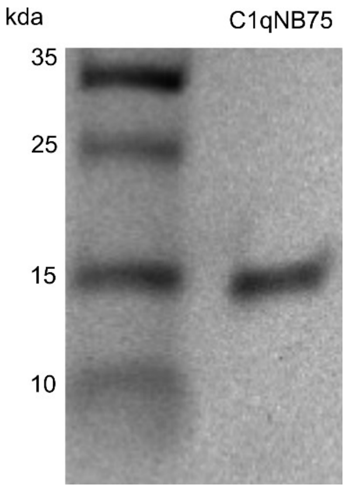

Figure S20. SDS-PAGE of C1qNB75 after purification. (PageRuler™ Plus Prestained Protein Ladder, 10 to 250 kDa (Themo Scientific™) was used as a reference. See Table S1 for the amino acid sequence of the construct.

Table S1: amino acid sequences of the protein constructs.

	Amino acid sequence
SC-gC1q	MARPLCTLLLLMATLAGALAGSDQPRPAFSAIRRNPPMGGNVVIFDTVITNQEEPYQNHSG<br>RFVCTVPGYYYFTFQVLSQWEICLSIVSSSRGQVRRSLGFCDTTNKGLFQVVSGGMVLQLQ<br>QGDQVWVEKDPKKGHIYQGSEADSVFSGFLIFPSAGSGKQKFQSVFTVTRQTHQPPAPNSL<br>IRFNAVLTNPQGDYDTSTGKFTCKVPGLYYFVYHASHTANLCVLLYRSGVKVVTFCGHTSK<br>TNQVNSGGVLLRLQVGEEVWLAVNDYYDMVGIQGSDSVFSGFLLFPDGSAKATQKIAFSAT<br>RTINVPLRRDQTIRFDHVITNMNNNYEPRSGKFTCKVPGLYYFTYHASSRGNLCVNLMRGR<br>ERAQKVVTFCDYAYNTFQVTTGGMVLKLEQGENVFLQATDKNSLLGMEGANSIFSGFLLFP<br>DMEAAAWSHPQFEKGAAWSHPQFEKGAAWSHPQFEKGAA*
C1qNB75	QVQLVETGGGLVQAGGSLRLSCAASGRTFNNDVMAWFRQAPGTEREFVALITAGGGTHYAD<br>SVKGRFVISRDNDKNMAYLQMNSLKSEDTAIYYCGADENPPGWPSRWSSAYDYWGQGTQVT<br>VSSHHHHHH*

Table S2: amino acid sequences and modifications on the peptides. * denotes the peptide is cyclized by means of chloroacetylation of the N-terminus. The lysine at the c-term position is either modified with an azide (Az) or a biotin (Bt). ‘ denotes the D-isomer of the tyrosine and is only used in the inv-cL3 peptide.

	Amino acid sequence
cL3-Az	YTVTFYPDPFTLQFIAC*GS{lys(N3)}G
cL3-Bt	YTVTFYPDPFTLQFIAC*GS{lys(Bt)}G
inv-cL3	Y’TVTFYPDPFTLQFIAC*GS{lys(Bt)}G
lin-L3	YTVTFYPDPFTLQFIASGS{Lys(Bt)}G
cL3-delN1	Y-VTFYPDPFTLQFIAC*GSK{Lys(N3)}
cL3-delN2	Y—-TFYPDPFTLQFIAC*GSK{Lys(N3)}
cL3-delN5	Y-----PDPFTLQFIAC*GSK{Lys(N3)}
cL3-delN6	Y------DPFTLQFIAC*GSK{Lys(N3)}
cL3-delN8	Y--------FTLQFIAC*GSK{Lys(N3)}
cL3-delC1	YTVTFYPDPFTLQFI-C*GSK{Lys(N3)}
cL3-delC2	YTVTFYPDPFTLQF—-C*GSK{Lys(N3)}
cL3-delC4	YTVTFYPDPFTL----C*GSK{Lys(N3)}
cL3-delC6	YTVTFYPDPF------C*GSK{Lys(N3)}

Table S3: Theoretical mass and HRMS data of each peptide. Ppm diff is determined by taking the observed m/z displayed in the column: HRMS (m/z) subtracting the appropriate theoretical m/z value either plus 2 or 3 protons and dividing by the theoretical value to quantify the difference between observed and theoretical m/z values.

	theoretical MW	*(M+2H)/2*	*(M+3H)/3*	*obsv Mass*	*ppm diff*
cL3-Az	2420.1147	1211.0652		1211.0627	2.06E-06
cL3-Bt	2620.2018		874.4084	874.4066	2.06E-06
inv-cL3	2620.2018		874.4084	874.4560	5.44E-05
lin-L3	2564.2297	1283.1227		1283.1191	2.81E-06
cL3-delN1	2391.3100	1196.0781		1196.0751	2.51E-06
cL3-delN2	2291.0721	1146.5440		1146.5400	3.49E-06
cL3-delN5	1879.8927	940.9542		940.9525	1.81E-06
cL3-delN6	1782.8399	892.4280		892.4262	2.02E-06
cL3-delN8	1570.7602	786.3874		786.3866	1.02E-06
cL3-delC1	2420.1511	1211.0834		1211.0796	3.14E-06
cL3-delC2	2307.0670	1154.5415		1154.5387	2.43E-06
cL3-delC4	2031.9400	1016.9780		1016.9754	2.56E-06
cL3-delC6	1817.8083	909.9121		909.9097	2.64E-06

Table S4: Sequences of the selected peptides from the RaPID screen. Data is provided in fasta format. Abbreviation gC1q-L-tyr-#1_#2 : gC1q corresponds to SCgC1q used in the screen, L-tyr corresponds to the chloro-acetylated tyrosine (L-stereoisomer), #1 corresponds to the position in the list and #2 corresponds to the relative abundance percentage of each sequence within the sequencing data. See also data availability section of the manuscript for the full sequencing dataset or ref 59.

>gC1q-L-tyr-1_0.40126
YYFKYVPYPTGLYNVYCSGGGSS*
>gC1q-L-tyr-2_0.18737
YYWAGCPSPLSDSGYRCSGGGSS*
>gC1q-L-tyr-3_0.09057
YTVTFYPDPFTLQFIACSGGGSS*
>gC1q-L-tyr-4_0.08282
YYWAGCSNLYINCGSRCSGGGSS*
>gC1q-L-tyr-5_0.03826
YYWAGCPSPLSDSGYSCSGGGSS*
>gC1q-L-tyr-6_0.02821
YYWAGCHSPLSDSGYRCSGGGSS*
>gC1q-L-tyr-7_0.0176
YYWAGCPSPLRDSGYRCSGGGSS*
>gC1q-L-tyr-8_0.01275
YYTSKGPNPFCLLWRDCSGGGSS*
>gC1q-L-tyr-9_0.01045
YCFWISPLPKPEWFLDCSGGGSS*
>gC1q-L-tyr-10_0.00527
YYWAGCSNLYINCGSSCSGGGSS*
>gC1q-L-tyr-11_0.00519
YFLNLKPSPFNWWNNYCSGGGSS*
>gC1q-L-tyr-12_0.00513
YYWAGCSNLYINCSGGGSS*
>gC1q-L-tyr-13_0.00342
YWLQSRPNPFQIEELWCSGGGSS*
>gC1q-L-tyr-14_0.00339
YYWAGCHSPLSDSGYSCSGGGSS*
>gC1q-L-tyr-15_0.00336
YYFKYVPYPTGLYNVYCSGSGS*
>gC1q-L-tyr-16_0.00237
YMYKLAPYPGWLGHSICSGGGSS*
>gC1q-L-tyr-17_0.00222
YYWAGCPSPLRDSGYSCSGGGSS*
>gC1q-L-tyr-18_0.00179
YYFKYVPYPTGLYNVYCRGGGSS*
>gC1q-L-tyr-19_0.00172
YYWAGCPSPLSDSGYRCSGSGS*
>gC1q-L-tyr-20_0.00143
YCFKYVPYPTGLYNVYCSGGGSS*
>gC1q-L-tyr-21_0.00116

YNVKWYPDPWLLTNPGCSGGGSS*
>gC1q-L-tyr-22_0.00114
YYWAGCPSQLSDSGYRCSGGGSS*
>gC1q-L-tyr-23_0.00113
YYFKYVPYPTGLYNVYCSGGSS*
>gC1q-L-tyr-24_0.00113
YYWAGCSNLYINCGSSDCSGGGSS*
>gC1q-L-tyr-25_0.00109
YICLFQKVAWFSLDDACGSGSGS*
>gC1q-L-tyr-26_0.00105
YYFKYVPYPTGLYNVYCSGGGRS*
>gC1q-L-tyr-27_0.00096
YTVTFYPDPFTLQFIACSGSGS*
>gC1q-L-tyr-28_0.00089
YYWAGCPSPLSDSGYRCRGGGSS*
>gC1q-L-tyr-29_0.00089
YPFCLHPIPLDLYIYLCSGGGSS*
>gC1q-L-tyr-30_0.00085
YYWAGCPSTLSDSGYRCSGGGSS*
>gC1q-L-tyr-31_0.00081
YTVTFYPNPFTLQFIACSGGGSS*
>gC1q-L-tyr-32_0.00081
YYFKYVPYQTGLYNVYCSGGGSS*
>gC1q-L-tyr-33_0.00078
YYFKYVPYPTGLYNVY*SGGGSS*
>gC1q-L-tyr-34_0.00078
YWSHPQFEKENLYFQSMGCGGSGGS*
>gC1q-L-tyr-35_0.00075
YLCLNNKLSWTTVPSGCGSGSGS*
>gC1q-L-tyr-36_0.00073
YYFKYVPYPTGLYNVYCSGGGSR*
>gC1q-L-tyr-37_0.00071
YYWAGCPSPLSDRGYSCSGGGSS*
>gC1q-L-tyr-38_0.00071
YYWAGCHSPLRDSGYRCSGGGSS*
>gC1q-L-tyr-39_0.00069
YYFKYVSYPTGLYNVYCSGGGSS*
>gC1q-L-tyr-40_0.00069
YYWAGCSNLYINCGSRCSGSGS*
>gC1q-L-tyr-41_0.00066
YYWAGCPFPLSDSGYRCSGGGSS*
>gC1q-L-tyr-42_0.00058
YYWAGCPSPLSDSGYRCSGGGRS*
>gC1q-L-tyr-43_0.00058
YYWAGCSNLYINCGSMCSGGGSS*
>gC1q-L-tyr-44_0.00058

YYWAGCPSPLSDSGYRCSGGSS*
>gC1q-L-tyr-45_0.00057
YYFKYVPYPTGLYNVYCNGGGSS*
>gC1q-L-tyr-46_0.00057
YYWAGCPSPLSDSGYR*SGGGSS*
>gC1q-L-tyr-47_0.00056
YYFKYVTYPTGLYNVYCSGGGSS*
>gC1q-L-tyr-48_0.00055
YYFKYVPYPTGLYNVYCSSGGSS*
>gC1q-L-tyr-49_0.00054
YYWAGCPSPLSDSGYGCSGGGSS*
>gC1q-L-tyr-50_0.00054
YWSHPQFEKENLYFQSMGCSGGGSS*
>gC1q-L-tyr-51_0.00053
YYFKYVPYPTGLYNVYCSGSGSS*
>gC1q-L-tyr-52_0.00053
YYFKYVPYPTGLYNVYCSGGSSS*
>gC1q-L-tyr-53_0.00053
YYFKYVPYPTGLYNVY*AAAAAA
>gC1q-L-tyr-54_0.0005
YYFKYVPYPNGLYNVYCSGGGSS*
>gC1q-L-tyr-55_0.00049
YYFKYVPYPTGLYNIYCSGGGSS*
>gC1q-L-tyr-56_0.00049
YYFKYVPYTTGLYNVYCSGGGSS*
>gC1q-L-tyr-57_0.00048
YYWDGCPSPLSDSGYRCSGGGSS*
>gC1q-L-tyr-58_0.00044
YYFKYVPYPTGLYNVCCSGGGSS*
>gC1q-L-tyr-59_0.00043
YKWFSDPNPFILAHYTCSGGGSS*
>gC1q-L-tyr-60_0.00042
YTCLSGRVSWSSWTPVCGSGSGS*
>gC1q-L-tyr-61_0.00041
YTVTFYPDPFTLQFIACRGGGSS*
>gC1q-L-tyr-62_0.00038
YQVFSFSQAIEEYCVWLQRRRSS*
>gC1q-L-tyr-63_0.00038
YYFKYVPYPTGLYNVYCSGGGS*
>gC1q-L-tyr-64_0.00038
YYWAGCLSPLSDSGYRCSGGGSS*
>gC1q-L-tyr-65_0.00037
YYWAGCTSPLSDSGYRCSGGGSS*
>gC1q-L-tyr-66_0.00036
YYWAGCSNLYINCGSRCRGGGSS*
>gC1q-L-tyr-67_0.00036

YICLAGRTAWARSESDCGSGSGS*
>gC1q-L-tyr-68_0.00036
YYWAGCPSPLSDSWYRCSGGGSS*
>gC1q-L-tyr-69_0.00036
YYFKYVPYPTGLYNVYCSGGGNS*
>gC1q-L-tyr-70_0.00036
YYWAGCPSPLSDSGYRCSGGGSR*
>gC1q-L-tyr-71_0.00033
YYWAGCSSPLSDSGYRCSGGGSS*
>gC1q-L-tyr-72_0.00033
YYWAGCSNLYINCGSRCSGGSS*
>gC1q-L-tyr-73_0.00033
YYWAGCASPLSDSGYRCSGGGSS*
>gC1q-L-tyr-74_0.00031
YYWAGCSNLYINCGYRCSGGGSS*
>gC1q-L-tyr-75_0.00031
YYFKYVPYPTGLYNVYCSGGDSS*
>gC1q-L-tyr-76_0.00029
YTVTFYPDPFTLQFIA*SGGGSS*
>gC1q-L-tyr-77_0.00029
YYWAGCPSPMSDSGYRCSGGGSS*
>gC1q-L-tyr-78_0.00029
YYWAGCSNLYINCGSRCSGGGRS*
>gC1q-L-tyr-79_0.00028
YYFKYVPYPTGLYNVYCSGGGSN*
>gC1q-L-tyr-80_0.00028
YYWAGCSNLYIYCGSRCSGGGSS*
>gC1q-L-tyr-81_0.00028
YTVTFYPDPFTLQFIECSGGGSS*
>gC1q-L-tyr-82_0.00028
YYFKYVPYPTGLYSVYCSGGGSS*
>gC1q-L-tyr-83_0.00027
YTVTFYPDPFTLQFIACSGGSS*
>gC1q-L-tyr-84_0.00027
YYFKYVPYPTGLYNVYYSGGGSS*
>gC1q-L-tyr-85_0.00027
YYFKYVPYPTGLYNV*CSGGGSS*
>gC1q-L-tyr-86_0.00026
YYWAGCPSPLSDSGYR*AAAAAA
>gC1q-L-tyr-87_0.00026
YYWAGCSNLYINCGSR*SGGGSS*
>gC1q-L-tyr-88_0.00025
YY*AGCPSPLSDSGYRCSGGGSS*
>gC1q-L-tyr-89_0.00025
YFYTWVPNPFVLGNLYCSGGGSS*
>gC1q-L-tyr-90_0.00024

YYWAGCPSPLSDSGYRCSGSGSS*
>gC1q-L-tyr-91_0.00023
YYWAGCSNLYINCGSRCSGGGSR*
>gC1q-L-tyr-92_0.00023
YYFKYVPYPTGLYNVYCSGGGTS*
>gC1q-L-tyr-93_0.00023
YTVTFYPDPFTLQFITCSGGGSS*
>gC1q-L-tyr-94_0.00023
YTVTFYPDPFTLQFIACSGGGRS*
>gC1q-L-tyr-95_0.00023
YYWAGCPYPLSDSGYRCSGGGSS*
>gC1q-L-tyr-96_0.00022
YYFKYVPYPTGLYNVYCGGGGSS*
>gC1q-L-tyr-97_0.00022
YYWAGCPSQLSDSGYSCSGGGSS*
>gC1q-L-tyr-98_0.00022
YYWAGCPSPLSDRGYRCSGGGSS*
>gC1q-L-tyr-99_0.00021
YYFKYVPYPTGLYNVYCSGGGGS*
>gC1q-L-tyr-100_0.00021
YKVTFYPDPFTLQFIACSGGGSS*
